# Precursor transformation and microbial responses during compost humification regulated by wood vinegar produced at different pyrolysis temperatures

**DOI:** 10.3389/fchem.2026.1912238

**Published:** 2026-10-21

**Authors:** Jia Liu, Muxi Zhao, Weiguo Xu, Siyu Li, Ziyang Xue, Gan Yang, Dongping Gao, Hui Yu, Xiaoyun Lian, Lingling Chen, Ling Zhou

**Affiliations:** 1 College of Mechanical and Electrical Engineering, Tarim University, Alar, China; 2 Modern Agricultural Engineering Key Laboratory at Universities of Education Department of Xinjiang Uygur Autonomous Region, Alar, China

**Keywords:** aerobic composting, humification, metagenomic analysis, spectroscopic techniques, wood vinegar

## Abstract

The humification stage is a critical that determines compost quality, and the addition of functional exogenous materials is an important measure to promote this stage. Cotton-stalk-derived wood vinegar, a bioactive liquid by-product of cotton stalk pyrolysis, may regulate compost humification by modulating organic matter transformation and microbial metabolism. However, the yield of these components is significantly influenced by the pyrolysis temperature. In this study, cotton stalk wood vinegar prepared at different pyrolysis temperatures was applied as an exogenous additive in co-composting with pig manure (T300, T400, T500, and T600). The regulatory effects of the compositional differences in wood vinegar on the transformation of humification precursors and microbial community succession were investigated, thereby elucidating the chemical–biological pathways of humus formation. The results showed that, compared with the control (CK), all wood vinegar treatments significantly enhanced the humification level (p < 0.05), with the T400 treatment achieving the highest humic acid content of 23.360 g/kg on day 15 of composting. Inter-component correlation analysis and FTIR two-dimensional correlation spectroscopy (2D-COS) revealed that the T400 treatment promoted the synergistic transformation of aromatic and nitrogenous precursors and accelerated condensation and polymerization reactions. Meanwhile, the T400 treatment exhibited the highest ratio of humic-like to protein-like fluorescence components, reaching 2.88:1. Regarding microbial community succession, the T400 treatment enriched microorganisms belonging to the phyla Pseudomonadota, Actinomycetota, and Bacillota, and enhanced carbon metabolism as well as glyoxylate and dicarboxylate metabolism activities. In conclusion, cotton stalk wood vinegar prepared at 400 °C effectively improved the degree of humification in the compost by promoting precursor transformation and optimizing the microbial environment. These findings provide a theoretical basis and practical guidance for the application of biomass-derived additives in composting.

## Graphical Abstract

**Figure:**
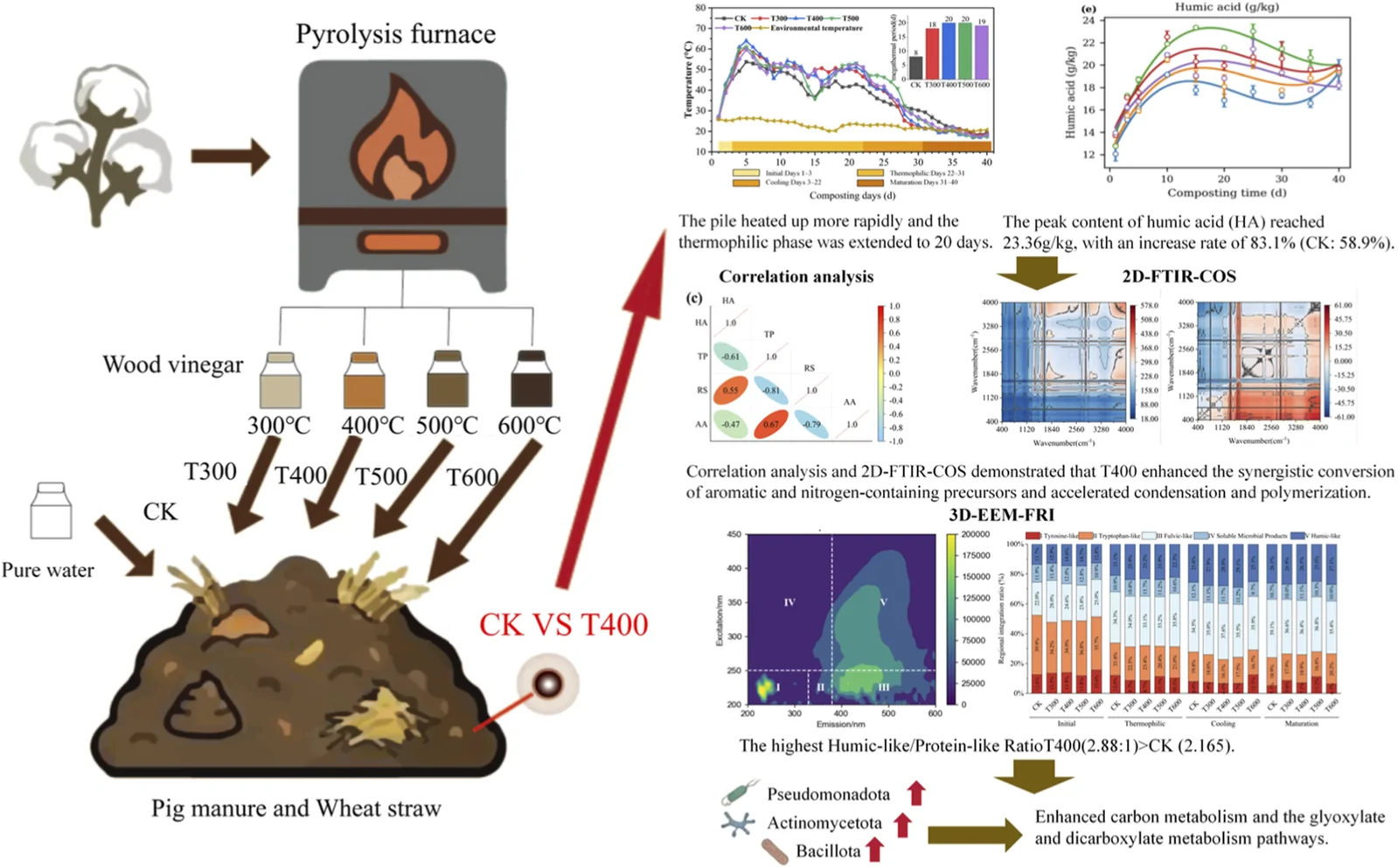

## 1 Introduction

The rapid accumulation of agricultural wastes worldwide has emerged as a critical challenge to environmental sustainability and the achievement of carbon neutrality goals (Wang F. et al., 2025). China provides a representative example: driven by the rapid expansion of agricultural and livestock industries, approximately 3.8 billion tons of livestock and poultry manure and nearly 0.9 billion tons of crop straw are generated annually (Wei et al., 2020). When improperly managed, these residues can release large quantities of malodorous and greenhouse gases, including NH_3_, H_2_S, and CH_4_, while nutrient losses via runoff lead to nitrogen and phosphorus enrichment in aquatic systems, triggering eutrophication. Moreover, the persistence of heavy metals and antibiotic residues poses long-term risks through bioaccumulation along the food chain (Qi et al., 2023). Collectively, these escalating environmental pressures underscore an urgent need for efficient and sustainable technologies for agricultural waste valorization.

Aerobic composting represents one of the most effective strategies for converting organic solid wastes such as straw and manure into stable, humus-rich fertilizers, thereby delivering substantial ecological and agronomic benefits. The degree of humification is widely recognized as the core determinant of compost quality (Ayilara et al., 2020). Fundamentally, humification is a complex, microbially driven biochemical process that involves the depolymerization of organic substrates, the transformation and condensation of intermediate products, and the eventual stabilization of macromolecular structures. The formation and release of humic acids and their key precursors—particularly polyphenolic compounds—are tightly constrained by feedstock characteristics, composting conditions, and microbial activity. These interacting factors collectively govern the rate and extent of humus formation and, ultimately, the functional quality of the compost product. Optimizing composting systems to accelerate humification and enhance humus yield therefore remains a central challenge in the field of organic waste resource recovery.

Recent studies have demonstrated that the introduction of functional additives—including organic amendments (e.g., biochar, humic precursors, and mature compost) and inorganic materials (e.g., zeolite and phosphate rock powder), and microbial inoculants can substantially alter the humification trajectory during composting (Lu M. et al., 2024; Su et al., 2024; Wang G. et al., 2022; Zhan et al., 2023; Zhang Z. et al., 2023). Consequently, elucidating how functional additives regulate precursor transformation and microbial community succession has become an active research focus in compost science in compost science (Shen et al., 2024).

To overcome the intrinsic limitations of conventional composting, diverse conditioners have been introduced to accelerate organic matter degradation and enhance humus condensation. Among these, biochar has attracted widespread attention due to its porous structure, strong adsorption capacity, and ability to provide microbial habitats. Importantly, the functional groups and physicochemical properties of biochar are highly dependent on pyrolysis temperature (Chen et al., 2017; Khater et al., 2024). Wang et al. explored the influence of biochar derived at varying pyrolysis temperatures (450 °C, 550 °C and 650 °C) on nitrogen conversion and retention throughout the composting process. It was revealed that pyrolysis temperature is a critical parameter for controlling composting kinetics and modulating microbial functional activity (Wang H. et al., 2023).

In parallel, iron-based Fenton reagents have been increasingly employed due to their ability to generate reactive oxygen species (especially -OH) *in situ* through Fe(II/III)–peroxide reactions within composting matrices. These reactive species effectively disrupt lignocellulosic complexes and cleave aromatic structures, thereby increasing substrate bioavailability. Moreover, appropriate dosages of Fenton reagents have been shown to optimize microbial community composition, enhancing the abundance of actinomycetes and carbohydrate-degrading enzymes and promoting synergistic substrate–microbe interactions that accelerate compost maturation (Jiao et al., 2024; Li K. et al., 2022).

Similarly, manganese salts facilitate humification through their multivalent redox cycling capability (Mn(II)/Mn(III)/Mn(IV)) and their stimulatory effects on oxidative enzymes such as laccase and manganese peroxidase (MnP), as well as on key microbial taxa. In straw composting systems, MnSO_4_ has been widely used as an “enzyme inducer,” with studies demonstrating that MnSO_4_ addition significantly enhances humic substance (HS) formation by stimulating functional microorganisms, restructuring microbial interaction networks, and promoting the conversion of precursors into humic fractions (Mu et al., 2024; Zhao et al., 2025). Beyond redox-active additives, inorganic coagulants also play a pivotal role in humification. Polynuclear hydroxy complexes formed during coagulant hydrolysis exhibit strong charge neutralization and bridging flocculation abilities, enabling interactions with extracellular polymeric substances (EPS) in sludge flocs. These processes redistribute dissolved organic matter (DOM), shift the balance between release and polymerization, and ultimately reshape substrate availability and metabolic preferences of key functional microbial groups, thereby promoting humus formation (Zhang Y. et al., 2024). Likewise, lime and phosphate amendments selectively drive organic matter toward humification pathways by elevating system pH, modifying reaction environments, and regulating the relative supply balance of C-P-N within composting matrices (Cai et al., 2019; Su et al., 2024).

The introduction of functional additives—including organic amendments (e.g., biochar, humic precursors, and mature compost) and inorganic materials (e.g., zeolite, and phosphate rock powder), and microbial.

Unlike the amendments discussed above, which are predominantly physical, mineral, or biological in nature, wood vinegar, also known as pyroligneous acid, is a liquid by-product of biomass pyrolysis and contains a complex mixture of organic compounds, including organic acids, phenols, ketones, and furans (Ouattara et al., 2023). In principle, wood vinegar can serve both as a readily bioavailable carbon source and as a direct precursor for humification. However, existing studies have often treated wood vinegar as a chemically homogeneous additive. For example, the combined addition of 10% biochar, 10% zeolite, and 2% wood vinegar reduced NH_3_ losses by 74.32% during pig manure composting (Wang et al., 2018), whereas the synergistic application of biochar and wood vinegar decreased greenhouse gas emissions by 61.9% and nitrogen losses by 35.8% (Wang et al., 2022d). Such an assumption may obscure a major source of functional variability, because pyrolysis temperature substantially alters the relative abundance of organic acids and phenolic compounds, thereby regulating the physicochemical and biological properties of wood vinegar (Lu et al., 2019). Pyrolysis temperature not only determines the relative proportions of acids, phenols, and alcohols in wood vinegar but also affects its pH and organic acid content (Ankona et al., 2023). Accordingly, the present study systematically compares wood vinegar produced at different pyrolysis temperatures and elucidates how its temperature-dependent chemical characteristics differentially regulate organic matter transformation, humus formation, and microbial community succession during composting. This temperature-gradient-based analytical framework distinguishes the present study from conventional wood vinegar amendment experiments and provides a mechanistic basis for the targeted production and application of wood vinegar in composting systems.

Against this backdrop, the present study systematically investigates the regulatory effects of cotton-stalk-derived wood vinegar produced under a gradient of pyrolysis temperatures (300–600 °C) on humification during aerobic composting. The core novelty of this study lies in the gradient comparison of temperature-dependent wood vinegar functions and the multi-technique coupled clarification of microbial mechanisms driving compost humification. We hypothesize that wood vinegar generated at an optimal pyrolysis temperature possesses a specific chemical composition capable of reshaping microbial metabolic networks, thereby synergistically promoting the transformation of reducing sugars and amino acids into humic substances.

Accordingly, this study is structured around three specific objectives: (I) to compare the humification-promoting effects of wood vinegar produced at different terminal pyrolysis temperatures and identify the optimal temperature range; (II) to elucidate the dynamic evolution of key humification precursors under wood vinegar amendment and reveal the chemical pathways facilitating humic substance synthesis; (III) to clarify the regulatory effects of wood vinegar on microbial community succession and microbial correlation networks.

## 2 Materials and methods

### 2.1 Composting experiment

Pig manure, wheat straw, and cotton stalks used in this experiment were collected from the 12th Regiment, Alar City, First Division of the Xinjiang Production and Construction Corps, China (40°30′32″N, 81°18′20″E). The cotton stalks were pyrolyzed in a carbonization furnace at a heating rate of 10 °C/min to final temperatures of 300, 400, 500, and 600 °C, respectively, and maintained under anaerobic conditions for 2.5 h to obtain cotton-stalk-derived wood vinegar at the corresponding temperatures. Prior to the experiment, the physicochemical properties of the pig manure were analyzed (Supplementary Table S1). Building upon previous studies conducted in our laboratory, wood vinegar obtained at 300 °C, 400 °C, 500 °C, and 600 °C, as well as deionized water, were each added to the composting mixtures at a concentration of 0.65% (volume-to-volume, V/V; 650 mL wood vinegar per 100 L compost matrix). The core physicochemical and compositional traits of cotton-stalk pyroligneous liquor exhibited clear temperature-dependent gradients (detailed raw data are provided in Supplementary Tables S2 and S3). The pH value of pyroligneous liquor gradually rose from 4.21 (300 °C) to 4.66 (600 °C) with increasing pyrolysis temperature. Phenolic compounds reached the maximum relative content of 34.64% at the medium temperature of 400 °C; lower-temperature (300 °C) liquor contained abundant phenolic products derived from lignin decomposition, while phenolic substances underwent secondary cracking, deoxygenation, and polymerization under high-temperature conditions, leading to a continuous decline in phenol proportion to 22.74% at 600 °C. The relative proportion of organic acids displayed a non-monotonic changing trend: organic acids slightly increased from 3.86% at 300 °C to 4.14% at 400 °C, then dropped to the minimum value of 2.67% at 500 °C due to intense decarboxylation and molecular cracking reactions. At 600 °C, severe secondary pyrolysis generated abundant low-molecular organic acids, which were enriched in the condensate and pushed the relative content of acids sharply up to 8.01%. Ketones and esters accumulated prominently at medium-high temperatures, with their peak proportions both observed at 500 °C (ketones: 19.33%, esters: 20.75%). Low-temperature pyroligneous liquor was rich in organic acids, medium-temperature (400 °C) liquor maintained a moderate phenolic content, and severe polymerization of phenolic fractions occurred under high temperature. This thermal-gradient-induced compositional variation is a key driver of the differences in humification performance among treatments. The physicochemical properties and composition of wood vinegar are presented in Supplementary Tables S2, S3. The moisture content of the composting piles was adjusted to 55%–65%, and the corresponding treatment groups were named T300, T400, T500, T600, and CK. Specifically, the CK group was supplemented with an identical volume of deionized water to offset solvent interference across all treatments. The C/N ratios of all five compost piles (T300, T400, T500, T600, CK) were subsequently determined and all remained between 25 and 30:1. Composting was carried out in self-made aerated composting boxes (60 × 45 × 35 cm), with each box loaded with an initial fresh material weight of 33 kg. The composting system adopted natural ventilation without forced aeration, and the piles were turned every 5 days to ensure sufficient oxygen supply. Samples were collected at the following time points: 0, 3, 5, 10, 15, 20, 25, 30, 35, and 40 days. The collected compost samples were divided into two parts: one portion was frozen at −20 °C for subsequent analysis of humification degree and spectral characteristics, while the other was stored at −80 °C for microbial metagenomic analysis. All physicochemical and spectral analyses were performed in triplicate; these replicates were technical rather than biological.

This study was conducted as fundamental research on laboratory-scale pig manure and wheat straw composting under the specified experimental conditions. Although the findings cannot be directly extrapolated to industrial-scale composting systems or composting processes involving other feedstocks, they provide important mechanistic insights and a scientific basis for process optimization, scale-up, and future engineering applications.

### 2.2 Physicochemical properties and analysis of humic substances and their precursors

The temperature at the central region of each compost pile was measured at three different locations every 24 h, and the average value was recorded. Fresh compost samples were extracted by shaking with deionized water (material-to-water ratio 1:10, w/v) at 25 °C and 180 rpm for 1 h. After settling and filtering, the pH value was determined using a pH meter. Reducing sugars (RS) were extracted using deionized water (1:5, w/v) at 80 °C for 40 min, and after cooling, the extract was centrifuged at 8,000 rpm for 10 min. The supernatant was then analyzed for its reducing sugar content using the 3,5-dinitrosalicylic acid (DNS) colorimetric method. The amino acid concentration was determined by the ninhydrin colorimetric method, following the protocol outlined (Wu J. et al. (2020). Polyphenolic compounds in freeze-dried samples were quantified using the Folin-Ciocalteu reagent colorimetric method (Jiang et al., 2021). The extraction and determination methods for HS and HA were based on the procedure reported by Zhou et al. (2014). The content of fulvic acid (FA) was calculated by subtracting the HA content from the total HS content.

### 2.3 Fourier transform infrared spectroscopy and three-dimensional fluorescence spectroscopy analysis

Compost samples (1 mg) were mixed with KBr powder in a ratio of 1:100 (w/w) and pressed into pellets under a pressure of 10 MPa for 0.5 min. Fourier transform infrared (FTIR) spectroscopy analysis was performed using a Nexus 870 FTIR spectrometer (Thermo Scientific, United States), with a scanning range of 4,000-400 cm^−1^. Each sample was scanned 32 times, with a resolution of 4 cm^1^. The FTIR spectra were analyzed to examine the changes in functional groups within the compost samples. Additionally, two-dimensional correlation spectroscopy (2D-COS), including both synchronous and asynchronous analysis, was employed on the infrared spectral data, and semi-quantitative analysis was performed based on the key peak ratios (Yu et al., 2019).

Three-dimensional excitation-emission fluorescence spectroscopy (3D-EEM) analysis was conducted using a PerkinElmer FL 8500 fluorescence spectrometer (United States) to investigate the changes in HS during the composting process. The compost samples were freeze-dried, dissolved in deionized water (1:10, w/v), and shaken for 24 h. The pH was adjusted to 7, and the solution was then diluted and filtered through a 0.45 µm membrane. The total organic carbon (TOC) concentration was maintained below 10 mg L^−1^. The excitation wavelength range was set at 200–450 nm (with a step size of 10 nm), and the emission wavelength range was 200–600 nm (with a step size of 1 nm). The scan speed was 1,200 nm min^−1^, and both excitation and emission slit widths were set to 5 nm. The spectral data were analyzed using region integration techniques (Zhu et al., 2024).

### 2.4 Metagenomic sequencing and DNA extraction

Samples collected on days 3, 10, 25, and 40 were subjected to metagenomic analysis after undergoing preprocessing prior to shipment. Total genomic DNA was extracted from compost samples using the FastPure Stool DNA Isolation Kit (Magnetic bead) (MJYH, Shanghai, China), following the manufacturer’s instructions. Post-extraction, the DNA concentration and purity were assessed, and DNA integrity was evaluated via 1% agarose gel electrophoresis. Qualifying samples were stored on dry ice and transported to Megi BioMed Technology Co., Ltd. (Shanghai, China) for library construction and metagenomic sequencing. DNA fragmentation was performed using the Covaris M220 (Gene Company, China), selecting approximately 350 bp fragments for the construction of paired-end (PE) libraries. Sequencing was performed on an Illumina NovaSeq™ X Plus (Illumina, United States) platform, employing the PE150 strategy. The non-redundant gene set was constructed using CD-HIT (v4.7, 90% similarity), and gene abundance was calculated using SOAPaligner (v2.21). The non-redundant gene set was then annotated for taxonomy and functional categories by comparison with the NR, KEGG, and COG databases (DIAMOND, e-value ≤ 10^−5^). Differential analysis was performed using the Kruskal-Wallis rank-sum test (Li X. et al., 2023).

### 2.5 Statistical analysis

All figures were generated using Origin 2024 software, and data analysis was conducted using Excel 2018. One-way ANOVA was performed using IBM SPSS 26 (IBM Corporation, United States) to evaluate significant differences in mean values between different treatment groups. Significant differences among the sample means were determined using SPSS 26. software. Fourier-transform infrared spectroscopy (FTIR) peak values were calibrated and analyzed using OMNIC 9.2 software. For FTIR analysis, all original transmittance spectra were baseline-corrected and normalized, then converted to absorbance (A = -lgT). Characteristic peak absorbance values were extracted to calculate FTIR intensity ratios for quantifying the humification degree and structural evolution of compost organic matter. Three-dimensional fluorescence spectroscopy data were analyzed using Python (Shu et al., 2025). The direct and indirect effects of compost environmental variables were analyzed using structural equation modeling. All data are presented as mean ± standard deviation (SD) from three independent experiments.

## 3 Results and discussion

### 3.1 Enhancement of composting temperature dynamics by wood vinegar amendment

During the composting process, temperature is a critical parameter that influences the decomposition rate of organic matter and microbial activity (Wang G. et al., 2022). As depicted in Figure 1, all treatment groups experienced a rapid increase in temperature on the first day of composting, entering the heating stage. This is likely due to the abundant availability of microbial carbon sources in the initial stage, leading to enhanced microbial activity (Mandpe et al., 2021). The temperature dynamics of wood vinegar treatment groups outperformed those of the control group (CK). In particular, the T400 and T500 groups exhibited a faster temperature rise relative to all other treatments. By Day 3, temperatures reached 50 °C, peaked above 60 °C on Day 5, and sustained the thermophilic range of 50 °C–65 °C for 20 days—longer than the 8-day thermophilic phase in the CK group. This suggests that the addition of wood vinegar can elevate the peak temperature of the composting process and prolong the high-temperature stage, which is typically associated with enhanced thermophilic microbial activity. This, in turn, accelerates the degradation of macromolecular organic matter such as cellulose, hemicellulose, and lignocellulose, while also aiding in the inactivation of parasites and pathogens (Wang L. et al., 2023; Wang et al., 2022b). This phenomenon may be attributed to the antimicrobial components present in wood vinegar, which effectively inhibit the growth of undesirable microorganisms and enhance the relative abundance of thermophilic bacteria. This result is also supported by subsequent microbial community analysis. After 22 days of composting, easily degradable small-molecule organic carbon was largely consumed, leaving behind predominantly recalcitrant organic matter. The continued depletion of available carbon sources suppressed microbial activity, leading to a reduction in heat production and a subsequent decline in the compost temperature, signifying the transition into the cooling and maturation stages of the composting process (Zhang et al., 2022). By Day 40, the temperatures in all treatment groups stabilized at ambient levels, indicating that the composting process had largely completed. From the observed temperature fluctuations, it is evident that wood vinegar prepared from temperatures of 400 °C and 500 °C accelerates the composting temperature rise and prolongs the high-temperature stage, thereby accelerating the decomposition of recalcitrant organic matter and providing more substrates for the humification process.

**FIGURE 1:**
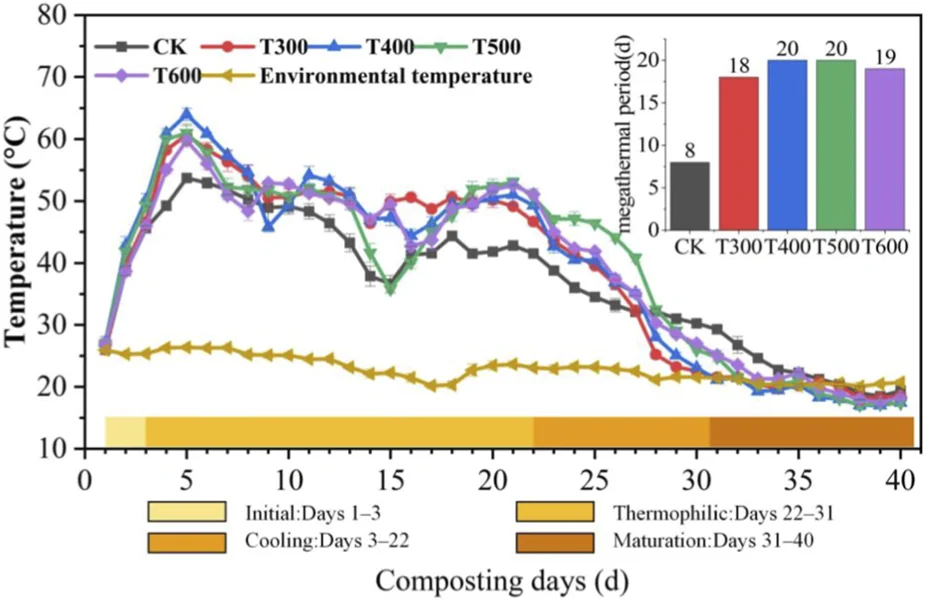
Temperature Variations of Five Compost Types at Different Stages of Composting (initial stage, days 1–3; thermophilic stage, days 3–22; cooling stage, days 22–31; maturation stage, days 31–40). The yellow line represents the ambient temperature. The inset graph illustrates the duration of the high-temperature stage.

### 3.2 Impact of wood vinegar amendment on precursor substances and humic acid formation

Humic precursor substances, including reducing sugars (RS), amino acids (AAs), and total phenols (TP), are continuously generated during composting through the hydrolysis of organic matter, structural breakdown, and microbial metabolic turnover. Simultaneously, they undergo various pathways such as the Maillard reaction, phenol-protein binding, and polyphenol self-condensation to form humic substances (Wu et al., 2024). Alternatively, they may be directly consumed by microorganisms as energy sources (Wu et al., 2017). The actual concentration of these precursors in the compost is determined by the dynamic balance between their generation and consumption rates, resulting in pronounced temporal fluctuations (Figure 2).

**FIGURE 2:**
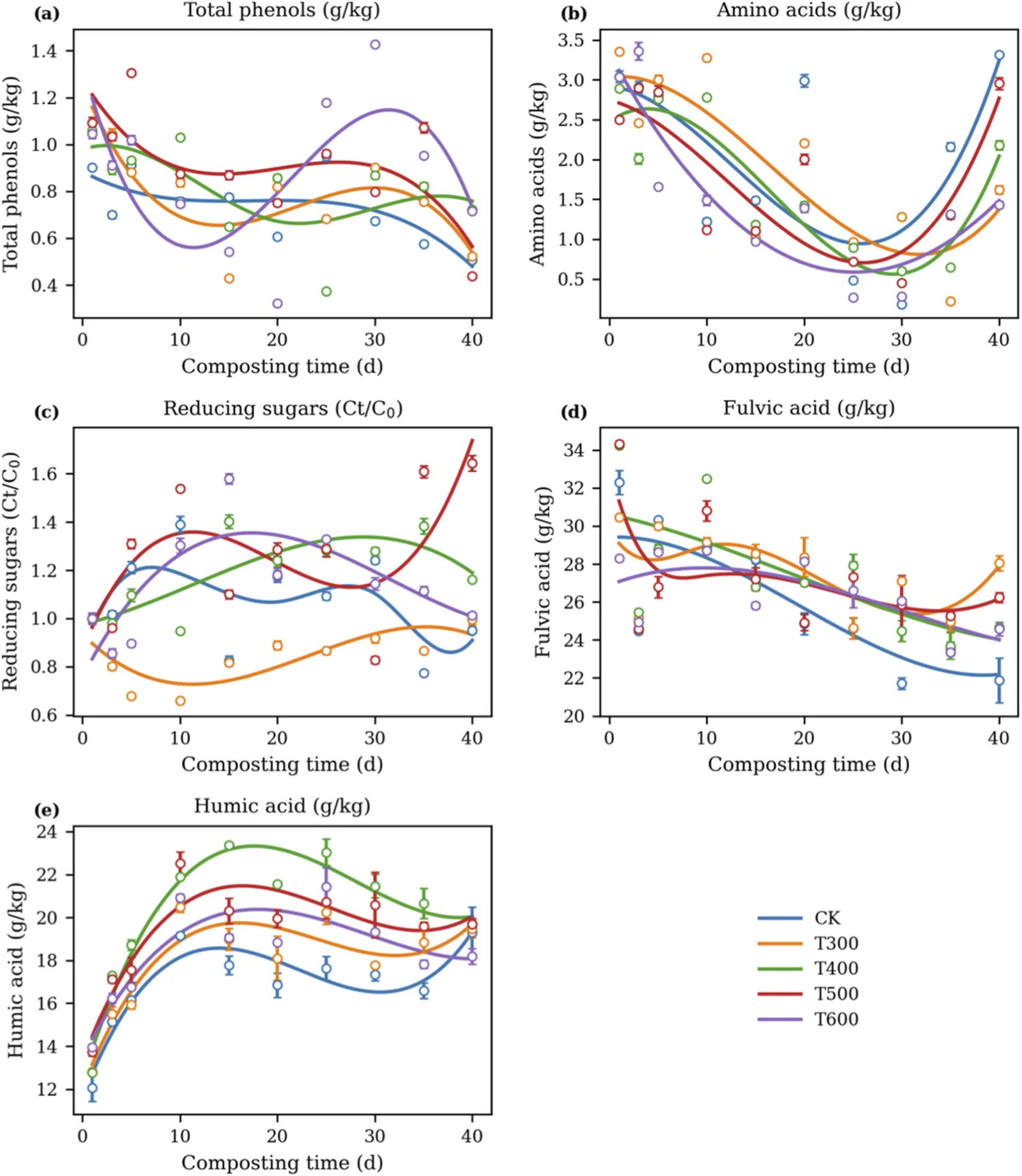
Changes in **(a)** total phenols, **(b)** amino acids, **(c)** reducing sugars (Ct/C_0_), **(d)** fulvic acid, and **(e)** humic acid during composting over time under five treatments (CK, T300, T400, T500, and T600). Data are expressed as means ± SD (n = 3). Solid lines represent LOESS smoothing followed by cubic spline fitting. Concentrations in panels **(a,b,d,e)** are expressed in g kg–1; Ct/C0 in panel **(c)** is dimensionless. Data are mean ± SD of three technical replicates.

The total phenol content in all treatment groups exhibited a general declining trend, with distinct mid-stage valleys and varying degrees of recovery in the later stages (Figure 2a). T400 and T600 decreased by 64.7% and 69.1% at Day 25 and Day 20, respectively, compared to their initial values, but both showed a rebound to above 0.71 g/kg at the end of the composting process. The decrease in T400 was more gradual, reflecting a “sustained transformation” rather than a “rapid depletion” in the early stage, which may have facilitated the involvement of phenolic compounds in the formation of HA. In contrast, the total phenol content in T500, T300, and CK declined more significantly, reaching values below 0.52 g/kg in the later stages, suggesting that the reduction of phenolic substances could be jointly driven by their participation in HA formation as humic precursors and microbial assimilation or degradation processes.

Amino acids (AAs) showed a pronounced decreasing trend during the first 30 days of composting (Figure 2b). Among the groups, the CK group exhibited the largest decrease, with a 94.0% reduction from the initial value, while T300 and T600 showed similar reductions of 93.2% and 91.1%, respectively. T400 demonstrated the smallest reduction, with only a 79.2% decline, indicating a more stable nitrogen precursor supply, potentially promoting the conversion of nitrogen sources into the humic framework or nitrogen-containing functional groups (Ma et al., 2022). The decline in amino acid concentrations arises from two coexisting processes: amino acids act as critical precursors involved in humification condensation reactions, and they are also consumed via microbial assimilation and biodegradation. The recovery of AAs in the later stages of composting has been reported in several studies, often related to microbial biomass turnover, cell lysis, and the redistribution of extractable nitrogen components (Yao et al., 2021).

Reducing sugars are highly degradable soluble carbon fractions, and their absolute contents are susceptible to differences in initial substrates. Therefore, Ct/C_0_ was adopted to represent the dynamic equilibrium between polysaccharide hydrolysis, release and consumption (Figure 2c). The Ct/C_0_ ratio in the CK group fluctuated consistently around 1.0, which may be attributed to the relatively low compost temperature and weak microbial activity, thereby maintaining reducing sugars in a dynamic equilibrium state. T400 reached a peak of 1.403 at 15 days, followed by a slight decrease, but still remained at 1.162 at 40 days, indicating that it could continuously provide sufficient carbon sources for compost microorganisms. In contrast, the Ct/C_0_ ratio of T300 was below 1 throughout the composting process and dropped to a minimum of 0.660 at 10 days, suggesting a potential shortage of carbon supply. T500 and T600 increased rapidly to above 1.5 on days 10 and 15, respectively. Subsequently, T600 showed a continuous decline, while T500 rose sharply in the later period, implying that T500 was more favorable for the accumulation of a soluble sugar pool during the maturation stage.

FA as an intermediate component in the humification process, generally showed a declining trend throughout the composting process (Figure 2d), indicating that FA is more likely to transform into more stable, aromatic HA structures rather than remaining as an independent end-product (Yang X. et al., 2023; Zhang et al., 2022). The greatest net decrease in FA content was observed in the CK group (32.3%), followed by T400 (28.0%), T500 (23.5%), and T600 (13.1%), with T300 showing the smallest decline at only 7.9% (Figure 2d). Moreover, T300 maintained the highest FA content at the end of composting, suggesting its tendency to accumulate small molecular humic components in the later stages. Despite the CK group experiencing the largest decrease, the fitting curves indicated that the FA content in all wood vinegar-treated groups remained higher than in CK throughout the composting process, indicating that the addition of wood vinegar promoted the generation of FA.

HA as the final product of the humification process, reflects the degree of humification in the composting system. As shown in Figure 2e, HA continuously accumulated during composting, with T400 exhibiting the most significant change. Its peak value reached 23.360 g/kg, representing an 83.1% increase, followed by T500 (64.0%), T300 (59.8%), and CK (58.9%), with T600 showing the smallest increase (53.7%). During the mid to later stages of composting, the concentration of humic acid (HA) peaked on the 15th day before exhibiting a declining trend. Previous research has indicated that the composition and structure of HA, as extracted by alkali during composting, undergo continuous changes. These changes are primarily characterised by a decrease in aliphatic components, a relative enrichment of aromatic components, an increase in oxygen-containing functional groups, and a rise in average molecular weight. It is likely that condensation and polymerisation reactions significantly contribute to the formation and structural reorganisation of humus (Sánchez-Monedero et al., 2002; Veeken et al., 2000). Furthermore, Gao et al. (2019) discovered that during the composting process, certain active organic components contribute to the formation of humin. This substance possesses a more intricate structure and enhanced stability, with its formation mechanism influenced by the composition of the composting raw materials and the transformation pathways of organic matter (Guo et al., 2019). We speculate that the reduction in HA content after the 15th day may be associated with the continued condensation, polymerisation, and structural reorganisation of humus. These processes could diminish the alkaline extractability of certain humic substances, leading to the redistribution of organic matter initially classified under the alkaline extraction of HA components into alkali-insoluble huminin-like components. The fitting curves revealed that HA content was consistently higher in wood vinegar-treated groups compared to CK throughout the composting process. Significance analysis of HA content during the compost maturity stage showed that, compared with the CK group, the T300, T400, T500, and T600 treatments significantly increased humic acid (HA) content (P < 0.05). Analysis of the significant differences in peak HA values further revealed that the T400 treatment exhibited the most pronounced effect among all treatments (P < 0.05), followed by T500, which was also significantly higher than the other groups (P < 0.05). No significant difference was observed between T300 and T600 (P > 0.05). All wood vinegar-treated groups differed significantly from the CK group (P < 0.05). These results indicate that wood vinegar addition promotes the accumulation of aromatic structural substances and the synthesis of HA, and that its effect is closely associated with the final pyrolysis temperature used for wood vinegar production, with wood vinegar produced at 400 °C showing the most significant humification-promoting effect (Fu et al., 2023; Huang et al., 2024).

In conclusion, the application of 400 °C wood vinegar significantly accelerated HA formation in the early to mid-stages of composting. The humification advantage of this treatment may be attributed to the concurrent stable supply of both carbon and nitrogen precursors and the efficient conversion of precursor substances into highly polymerized humic structures.

Through a comprehensive analysis of the dynamic variations in precursor substances and humic fractions, it was observed that the application of wood vinegar produced at different terminal pyrolysis temperatures markedly altered the humification characteristics during composting. The underlying transformation patterns were further elucidated by correlation analysis between precursor compounds and humic fractions (Ewert et al., 2018). It should be noted that correlation analysis only reflects the covariation patterns among variables and cannot determine the specific transformation pathways or directions of precursor substances, nor can it establish causal relationships. Therefore, the present results provide only associative evidence for the potential involvement of these factors in the humification process and should not be interpreted as direct proof of the underlying mechanisms. An integrated analysis across all treatments revealed that HA was generally negatively correlated with total phenols (TP) and amino acids (AA), while exhibiting a positive correlation with reducing sugars (RS). This pattern suggests that TP and AA may directly participate in HA formation, whereas RS likely facilitate HA accumulation indirectly by supplying energy for microbial metabolism and humification reactions. Notably, humification is not governed by a single precursor but rather depends on the synergistic transformation of multiple precursor pools (Wu et al., 2024). Distinct correlation patterns were observed among treatments. In the CK group, HA exhibited negligible correlations with TP, RS, and AA, indicating that, under untreated conditions, precursor substances failed to establish an effective synergistic interaction. In the T300 group, TP and AA displayed a strong positive correlation, while HA showed pronounced negative correlations with both TP and AA, providing supplementary evidence for a degree of covariation between aromatic and nitrogen-containing components in this treatment. In the T500 group, HA was strongly negatively correlated with AA, hinting at potential linkage implying that HA formation in this treatment may have relied more heavily on the consumption of nitrogenous compounds. However, both T300 and T500 showed weak correlations between HA and RS, suggesting a potential limitation in energy supply. In the T600 group, HA exhibited an extremely strong positive correlation with RS and a strong negative correlation with AA, whereas its correlation with TP was nearly negligible. This provides supplementary evidence that HA formation in this treatment was primarily driven by sugar-mediated nitrogen consumption, with comparatively limited involvement of phenolic precursors. In contrast to the above treatments, the T400 group demonstrated the most coordinated precursor interaction network. HA showed a moderate positive correlation with RS, while RS was strongly negatively correlated with both TP and AA. Meanwhile, TP and AA maintained a strong positive correlation. These relationships hint at potential linkage that, under sufficient carbon-derived energy supply, aromatic and nitrogen-containing precursors were synchronously transformed, thereby establishing a highly efficient humification pathway. This coordinated correlation structure is consistent with the process advantage previously observed in Figure 2e, where T400 exhibited rapid HA accumulation during composting and significantly higher HA levels compared to the CK group.

### 3.3 Fourier transform infrared spectroscopy analysis

Figure 3 illustrates the Fourier transform infrared (FTIR) spectra of compost samples from different treatments at various composting stages. All treatments exhibited characteristic infrared absorption features typical of organic matter. The broad band centered at 3,404 cm^−1^ is attributed to the stretching vibration of hydroxyl (O-H) groups, whereas the absorption peaks at 2,920 and 2,829 cm^−1^ correspond to the stretching vibrations of aliphatic -CH_2_/-CH_3_ groups. The band at 1,630 cm^−1^ is assigned to aromatic C=C or C=O vibrations, which are associated with aromatic structures and carboxylate functionalities (Demyan et al., 2012; Elakiya and Arulmozhiselvan, 2021). In addition, the absorption peaks at 1,356, 1,064, and 775 cm^−1^ are related to the bending vibration of phenolic O-H or C-O stretching, the C-O stretching of alcohols/ethers or polysaccharides, and the out-of-plane C-H deformation of substituted aromatic rings, respectively (Nuzzo et al., 2020).

**FIGURE 3:**
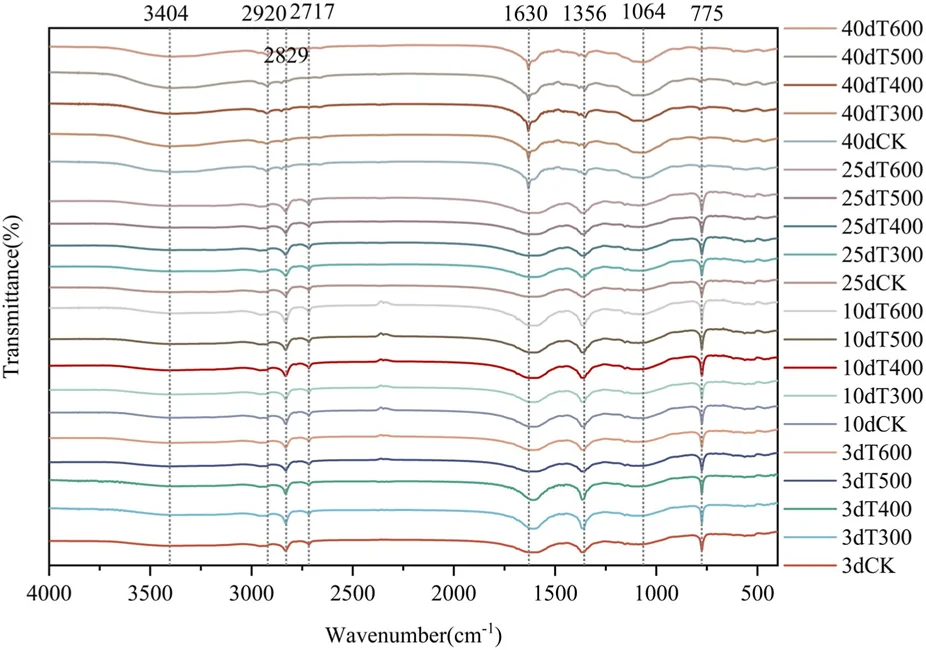
FTIR spectra showing the evolution of compost samples at different composting stages (3, 10, 25, and 40 days) under various treatments (CK, T300, T400, T500, and T600). The numbers at the top indicate characteristic peaks, including 3,404, 2,920, 2,717, 2,829, 1,630, 1,356, 1,064, and 775 cm^−1^. Spectra are plotted as transmittance (%) versus wavenumber (cm^−1^).

All raw transmittance FTIR spectra were preprocessed via baseline correction and unified normalization, and converted to absorbance (A = −lgT) for subsequent characteristic peak intensity ratio calculation in semi-quantitative analysis.

With increasing composting time, the aliphatic characteristic peaks at 2,920 and 2,829 cm^−1^ gradually weakened across all treatments, indicating the continuous degradation of labile organic matter (Lin et al., 2022). Notably, T400 and T500 exhibited a more pronounced decline in peak intensity during the initial composting stage (3–10 days), suggesting that these treatments enhanced microbial degradation of organic substrates, thereby facilitating the formation of low-molecular-weight intermediates and accelerating the humification process. During the mid-to-late composting stages (25–40 days), the absorption band at 1,630 cm^−1^ progressively intensified in all treatments, reflecting the accumulation of aromatic structures and humic substances (Liu et al., 2021; Sun et al., 2022). Among them, T400 demonstrated the most significant enhancement of aromatic features, with peaks becoming sharper and more stabilized, indicative of increased structural condensation and aromaticity. Although the absorption intensities at 775 and 1,356 cm^−1^ decreased over time in all treatments, this phenomenon does not imply a reduction in the degree of humification. Rather, it likely reflects progressive condensation and structural reorganization of aromatic and polar functional groups, leading to the formation of more complex and stable macromolecular humic substances (Cherepanov and Kryukova, 2020; Jouraiphy et al., 2008).

To further elucidate the degree of organic matter humification, the composting process under different wood vinegar treatments was systematically evaluated using FTIR-derived indices, including I_1630_/I_2920_, I_1630_/I_3400_, I_1630_/(I_2920_ + I_3400_), and I_1630_/I_1064_ (Abid et al., 2020; Silva et al., 2025). All peak intensity ratios were calculated based on baseline-corrected, normalized absorbance spectra converted from original transmittance data. As illustrated in Figure 4, during the initial stage (3–10 days), variations in these indices were relatively limited across treatments. However, following the principal reaction stage (10–25 days), the structural superiority of T400 gradually became evident. Specifically, its I_1630_/I_2920_ value increased markedly from 2.58 at day 10 to 7.85 at day 40, representing the largest increment among all treatments and indicating that aromatic condensation processes predominated during the later composting stage (Silva et al., 2025).

**FIGURE 4:**
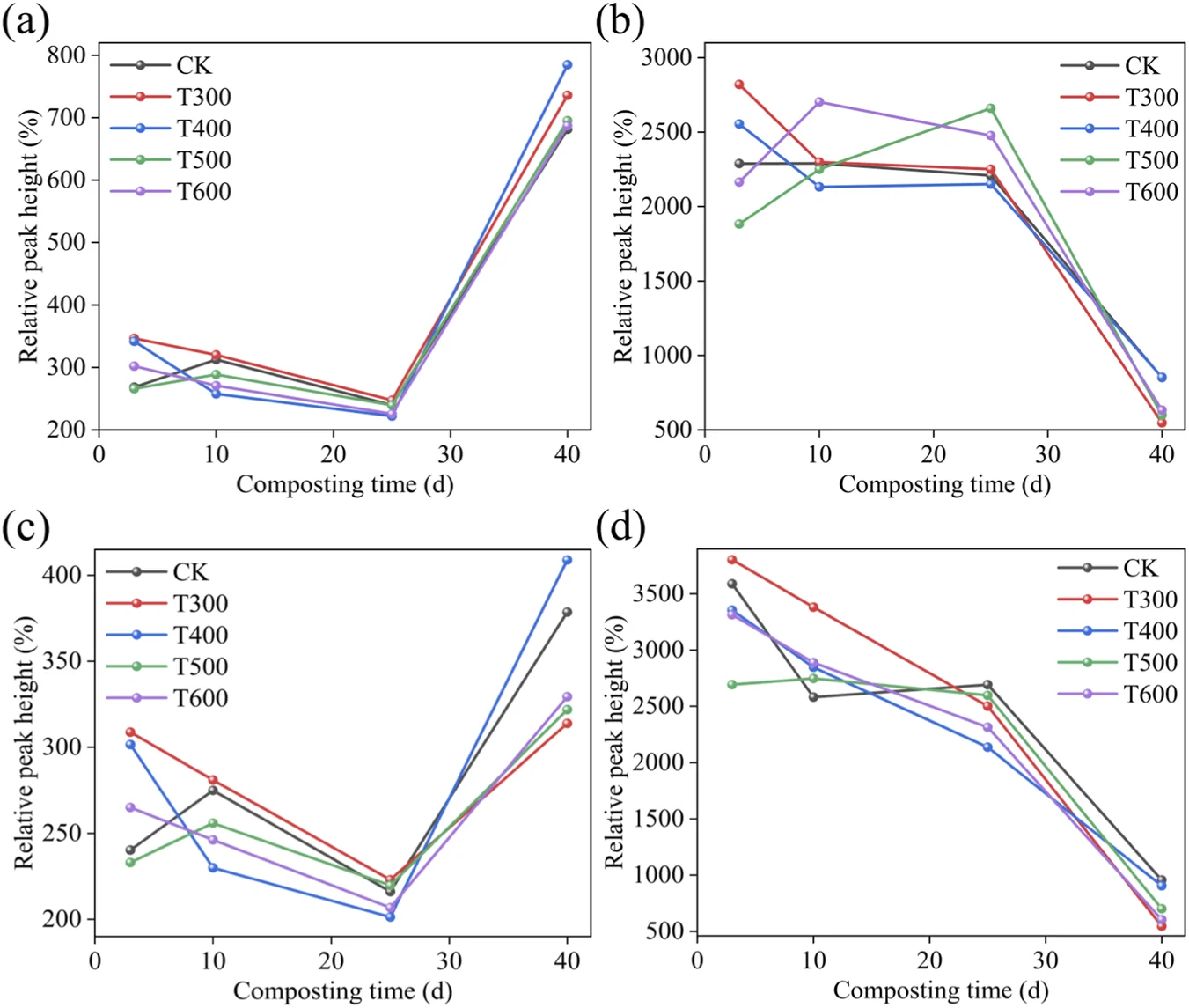
Variations in the FTIR peak intensity ratios during composting under different treatments (CK, T300, T400, T500, and T600): **(a)** I1630/I2920, **(b)** I1630/I3400, **(c)** I1630/(I2920 + I3400), and **(d)** I1630/I1064. All spectra were baseline-corrected, normalized, and converted from transmittance to absorbance (A = −lgT) before ratio calculation.

In contrast, T300 exhibited only a modest increase in I_1630_/I_2920_, suggesting a comparatively limited capacity to promote aromatization. Although T600 displayed a relatively high I_1630_/I_3400_ ratio at day 10 (27.02), its I_1630_/I_2920_ and I_1630_/(I_2920_ + I_3400_) values failed to remain elevated by day 40, implying insufficient structural stabilization of humified products. The evolution of I_1630_/(I_2920_ + I_3400_) and I_1630_/I_1064_ further corroborated these findings. Notably, T400 exhibited the greatest increase in I_1630_/(I_2920_ + I_3400_) during 25–40 days (+2.08), reaching 4.09 at day 40. Concurrently, its I_1630_/I_1064_ ratio remained comparatively high (9.05) at the end of composting. These results collectively indicate that T400 achieved superior efficiency in structural reorganization, aromatization, and stabilization of organic matter during humification (Aguelmous et al., 2020).

To elucidate the sequential variations of functional groups and thereby reveal how wood vinegar produced at different pyrolysis temperatures regulates the transformation pathways of key moieties-including polysaccharide C-O, amide/carbonyl, and aromatic structures-and their differential impacts on the molecular mechanisms of compost humification, the obtained spectral data were arranged according to composting time. Subsequently, two-dimensional correlation Fourier-transform infrared spectroscopy (2D-FTIR-COS) was conducted for each treatment. The synchronous and asynchronous maps within the wavenumber range of 4,000-400 cm^−1^ were generated (Wang D. et al., 2023) (Figure 5b), and twelve characteristic peaks were selected to determine their sequential order of variation (Supplementary Table S4).

**FIGURE 5:**
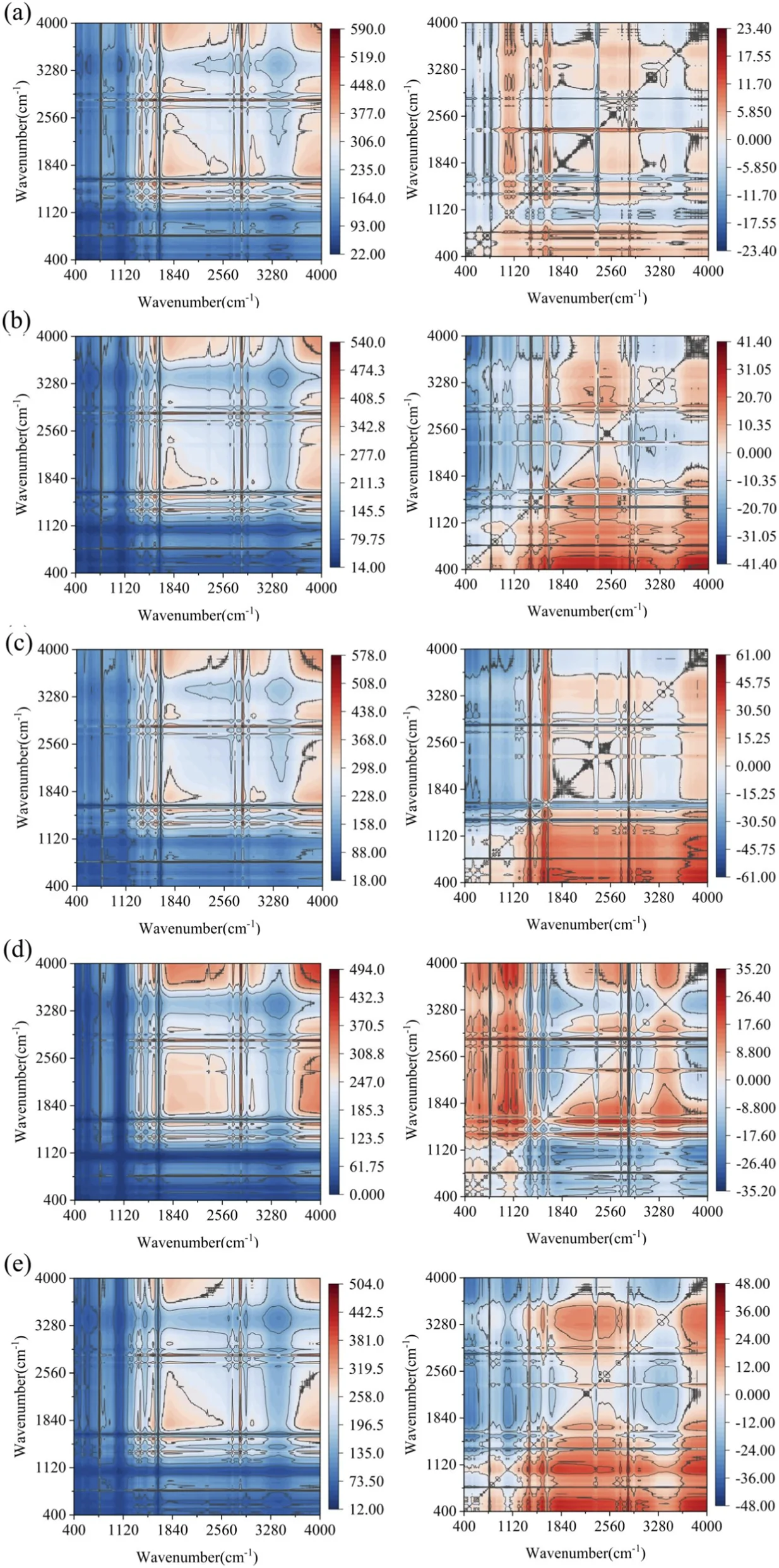
Synchronous (left) and asynchronous (right) FTIR contour plots for composting treatments under **(a)** CK, **(b)** T300, **(c)** T400, **(d)** T500, and **(e)** T600. The contour plots show the changes in intensity across different wavenumbers (400-4,000 cm^−1^) during the composting process. The color bars represent the intensity scale for each plot, with blue indicating lower intensities and red indicating higher intensities. The synchronous plots reflect the overall intensity variation over time, while the asynchronous plots display the stage shift between different wavenumbers.

The results demonstrated that the sequential order for the CK treatment was: 1,540 cm^−1^ > 1720 cm^−1^ > 1,630 cm^−1^ > 1,356 cm^−1^ > 2,829 cm^−1^ > 775 cm^−1^ > 2,717 cm^−1^ > 2,920 cm^−1^ > 3,404 cm^−1^ > 1,420 cm^−1^ > 1,270 cm^−1^ > 1,064 cm^−1^. This pattern indicates that nitrogen-containing components (1,540 cm^−1^) responded first, followed by carbonyl groups (1720 cm^−1^) and aromatic/conjugated structures (1,630 cm^−1^), whereas polysaccharides (1,064 cm^−1^) exhibited the latest variation. Such a sequence suggests insufficient carbohydrate-derived energy supply in the CK group, resulting in limited synergistic transformation between polyphenols and amino acids. This observation is consistent with the preceding correlation analysis, which revealed weak associations among reducing sugars, amino acids, and polyphenols, accompanied by a relatively slow humification process.

In the T400 treatment, the sequential order was: 1,064 cm^−1^ > 1,270 cm^−1^ > 1,420 cm^−1^ > 1,540 cm^−1^ > 1720 cm^−1^ > 2,920 cm^−1^ > 3,404 cm^−1^ > 1,630 cm^−1^ > 2,717 cm^−1^ > 1,356 cm^−1^ > 2,829 cm^−1^ > 775 cm^−1^. Here, polysaccharides (1,064 cm^−1^) exhibited the earliest variation, closely followed by phenolic/aromatic precursors (1,270 cm^−1^), while nitrogen-containing components (1,540 cm^−1^) also responded at an early stage. These findings indicate that under relatively sufficient carbon supply, the T400 treatment facilitated efficient synergistic humification between aromatic and nitrogenous precursors, thereby constructing a more coherent and effective humification pathway (Wang et al., 2024).

For the T300 treatment, the order was: 1,064 cm^−1^ > 1,630 cm^−1^ > 1,356 cm^−1^ > 1,540 cm^−1^ > 775 cm^−1^ > 2,829 cm^−1^ > 2,920 cm^−1^ > 1,420 cm^−1^ > 1,270 cm^−1^ > 1720 cm^−1^ > 3,404 cm^-1^ > 2,717 cm^−1^. Although carbohydrate mobilization (1,064 cm^−1^) occurred relatively early, peaks associated with carbonyl-related reactions (1720 and 2,717 cm^−1^) and phenolic/aromatic precursors (1,270 cm^−1^) appeared at later stages, suggesting ineffective coordination among key precursors. In contrast, the T500 treatment exhibited the following order: 1,356 cm^−1^ > 775 cm^−1^ > 1,540 cm^−1^ > 1,630 cm^−1^ > 2,829 cm^−1^ > 1720 cm^−1^ > 2,717 cm^−1^ > 1,420 cm^−1^ > 2,920 cm^−1^ > 1,270 cm^−1^ > 3,404 cm^−1^ > 1,064 cm^−1^. Notably, this pattern was nearly opposite to that of T300. Aromatic ring structures (775 cm^−1^) and nitrogen-containing components (1,540 cm^−1^) responded preferentially, whereas polysaccharides (1,064 cm^−1^) exhibited the latest variation. This implies that humification in the T500 group relied more heavily on amino acid (AA) consumption, with a comparatively weak association with reducing sugars (RS), consistent with the conclusion presented in Figure 6d that HA formation depended more strongly on AA depletion than on RS transformation.

**FIGURE 6:**
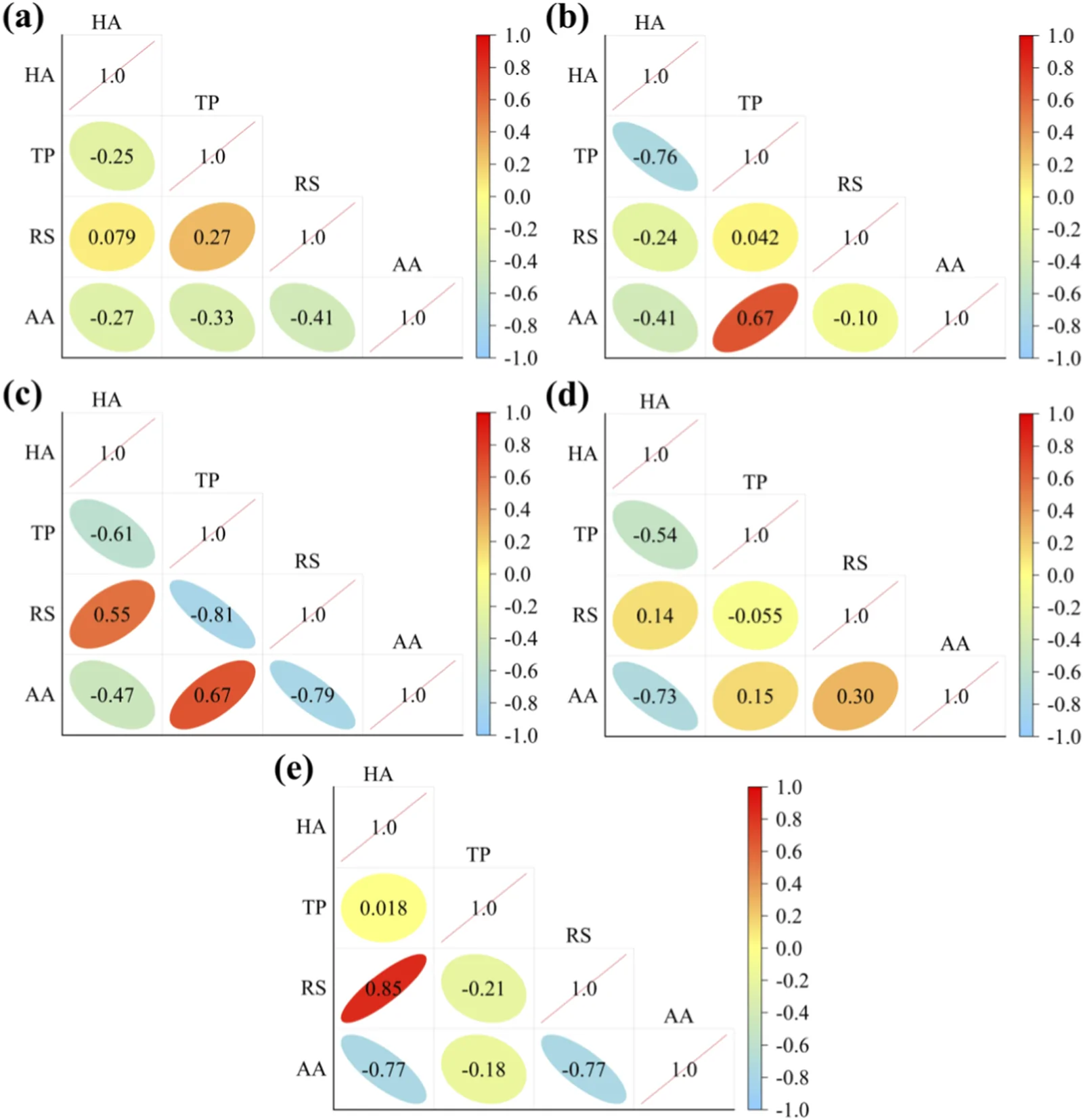
Pearson correlation analysis between HA, total phenols (TP), reducing sugars (RS), and amino acids (AA) during composting under **(a)** CK, **(b)** T300, **(c)** T400, **(d)** T500, and **(e)** T600 treatments. The color and shape of the ellipses represent the strength and direction of the correlation, with red indicating a positive correlation and blue indicating a negative correlation. Darker colors indicate stronger correlations. The numbers inside the ellipses show the corresponding correlation coefficients.

For the T600 treatment, the sequence was: 1,064 cm^−1^ > 3,404 cm^−1^ > 1,270 cm^−1^ > 1,420 cm^−1^ > 2,920 cm^−1^ > 1,630 cm^−1^ > 1,540 cm^−1^ > 1720 cm^−1^ > 1,356 cm^−1^ > 2,829 cm^−1^ > 2,717 cm^−1^ > 775 cm^−1^. Although polysaccharides (1,064 cm^−1^) and phenolic/aromatic precursors (1,270 cm^−1^) exhibited relatively early variations, the delayed response of nitrogen-containing components (1,540 cm^−1^) suggests that the overall synergistic transformation among precursors was still inferior to that observed in the T400 treatment. Collectively, the 2D-FTIR-COS results corroborate the aforementioned correlation analyses, demonstrating that the T400 treatment achieved the most coordinated synergistic evolution among diverse precursor substances during humification, thereby exerting a markedly superior promoting effect compared with the CK and other wood vinegar treatments. This phenomenon may be attributed to the abundance of phenolic compounds in wood vinegar produced at 400 °C (Oramahi et al., 2020). Such compounds not only facilitate the formation of aromatic structures but also promote synergistic transformations between phenolics and nitrogenous substances, ultimately establishing a more efficient humification pathway (Du et al., 2025).

### 3.4 Three-dimensional fluorescence spectroscopic analysis

Three-dimensional excitation-emission matrix (3D-EEM) fluorescence spectroscopy was employed to elucidate the compositional evolution of dissolved organic matter (DOM) during composting (Kong et al., 2023; Wang et al., 2024). The results are shown in Figure 7. Within the excitation (Ex = 200–450 nm) and emission (Em = 200–600 nm) wavelength domains, the fluorescence spectra were partitioned into five characteristic regions: tyrosine-like proteins (Region I), tryptophan-like proteins (Region II), fulvic acid-like substances (Region III), soluble microbial by-products (Region IV), and humic acid-like substances (Region V) (Li et al., 2023). The results revealed a progressive transition of DOM composition from protein-like constituents toward humified fractions throughout the composting process. In the initial stage, Regions I and II predominated, indicating the enrichment of readily biodegradable proteins and amino acid-derived substances. As composting advanced-particularly from the thermophilic to the cooling stage-the protein-like fractions continuously declined, whereas Regions III and V gradually became dominant. This shift reflects the increasing structural complexity and stabilization of DOM (Tai et al., 2024). Compared with the maturation stage, the cooling stage represents a critical transitional window from rapid biotransformation to structural stabilization, during which inter-treatment differences in humification rate and pathway become more pronounced (Wu et al., 2023). The humification parameter (HP), defined as (III + V)/(I + II) based on fluorescence regional integration (Peng et al., 2020), was employed to quantitatively assess the degree of humification. During the cooling stage, the HFRI values of T400 and T500 reached 2.880 and 2.662, respectively, significantly exceeding those of T300 (2.421), T600 (2.098), and CK (2.165). Concurrently, T400 and T500 exhibited the lowest residual proportions of protein-like components (I + II), at 22.7% and 24.2%, respectively. These findings indicate that these treatments more effectively facilitated the depletion of labile DOM and promoted its transformation into fulvic acid-like precursors and humic acid-like stable products.

**FIGURE 7:**
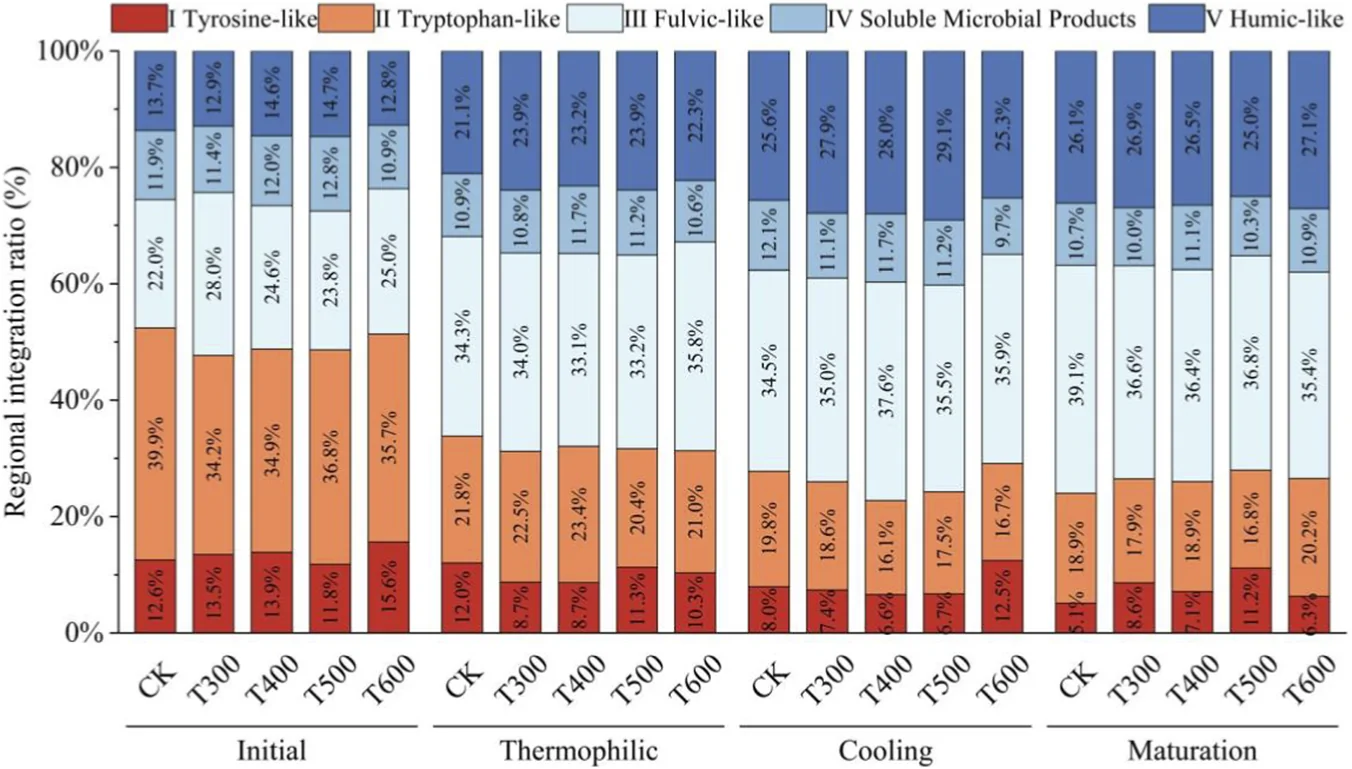
Regional integration ratios of different components (I: Tyrosine-like, II: Tryptophan-like, III: Fulvic-like, IV: Soluble Microbial Products, V: Humic-like) in the composting process under different treatments (CK, T300, T400, T500, and T600). The ratios are shown for each stage: Initial stage, thermophilic stage, cooling stage, and maturation stage. The stacked bars represent the distribution of the different components as percentages of the total integration ratio at each stage of composting.

In contrast, the promotive effect of T300 appeared relatively delayed. For T600, the elevated pyrolysis temperature may have induced excessive aromatic condensation of organic constituents, thereby suppressing microbial activity and constraining the conversion of humification precursors. Although HFRI values among treatments tended to converge or rebound during the maturation stage, such changes do not necessarily imply humification regression. Previous studies have reported a resurgence of protein-like fluorescence signals in the late or mature stage, likely attributable to sustained microbial metabolism and the production of extracellular enzymes and polymeric substances contributing to proteinaceous DOM (Kong et al., 2023; Tai et al., 2024; Wang S. P. et al., 2022). Taken together, the compositional succession patterns and quantitative fluorescence indices consistently support the ranking of humification-promoting efficacy as T400 ≈ T500 > T300 > T600 > CK. At the molecular level, the 3D-EEM results further elucidate the intrinsic linkage between precursor transformation dynamics and the formation of HA and FA, providing mechanistic insight into the differential regulatory effects of wood vinegar treatments on compost humification.

### 3.5 Effect of wood vinegar addition on the evolution of microbial community structure in composting

#### 3.5.1 α-diversity and β-diversity of microbial communities

α-Diversity analysis was employed to assess microbial diversity during the composting process (Figure 8). The results of the Chao1 and Shannon indices revealed a distinct differentiation in microbial diversity from the heating stage onward, with a further exacerbation of α-diversity divergence as the process entered the high-temperature stage. Compared to the CK treatment, the species richness and diversity of both bacteria and fungi were reduced in the T400 and T500 treatments, with a more pronounced decline observed in T400, particularly in the fungal community. This reduction could be attributed to the relatively stable bacterial community structure in the T400 treatment, where the high-temperature environment selectively enriched thermophilic microorganisms involved in organic matter mineralization and humification (Liang et al., 2021). During the cooling and maturation stages, α-diversity gradually stabilized across all treatments, indicating that the microbial community underwent restoration and reorganization following high-temperature selection (Zhong et al., 2020).

**FIGURE 8:**
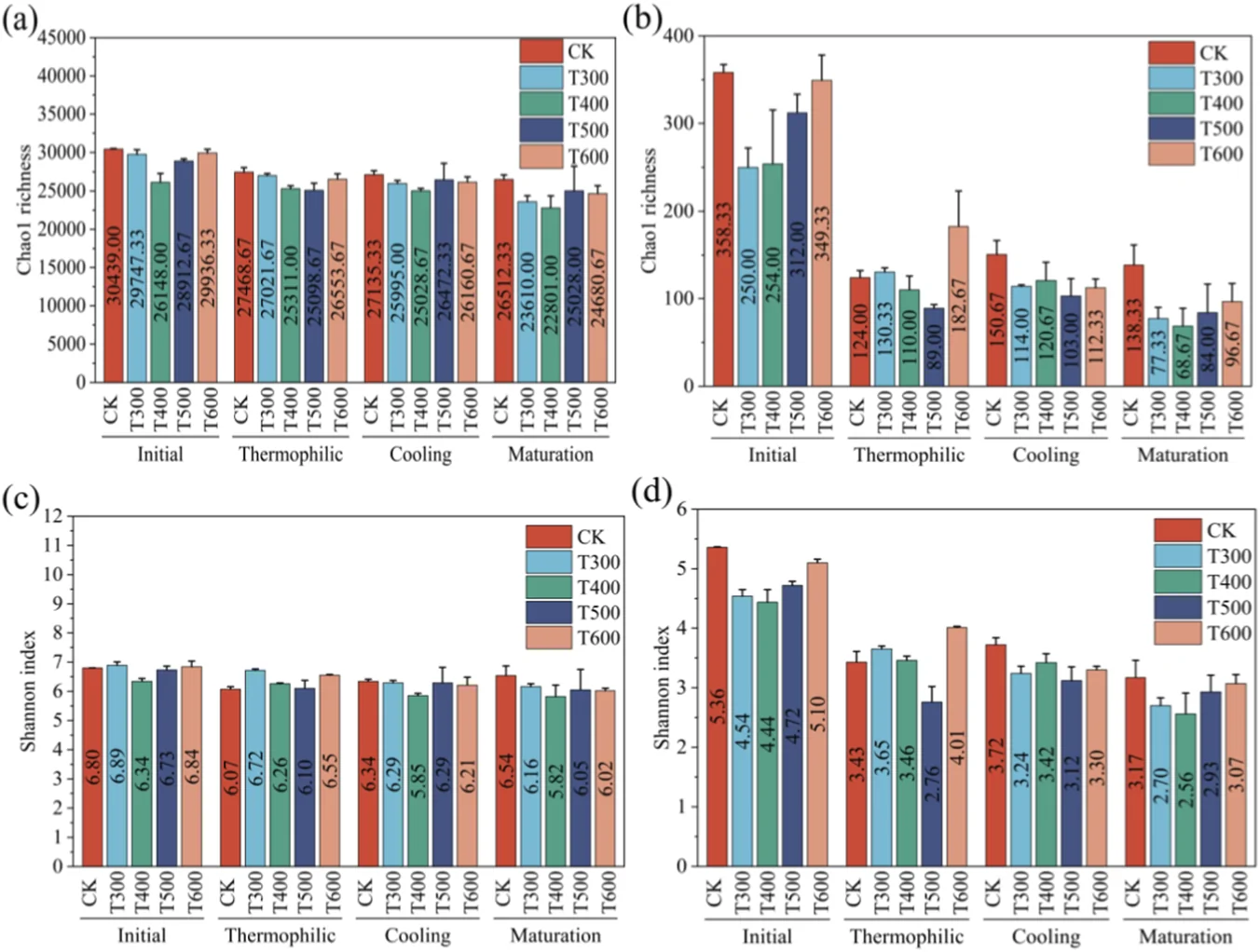
Chao1 and Shannon indices at the species level for bacteria and fungi during composting under different treatments (CK, T300, T400, T500, and T600) across the Initial, thermophilic, cooling, and maturation stages. **(a)** Bacterial Chao1 index, **(b)** Fungal Chao1 index, **(c)** Bacterial Shannon index, and **(d)** Fungal Shannon index.

As shown in Figure 9, beta-diversity analysis demonstrated significant differences in microbial community structures among the experimental treatments, indicating that wood vinegar application continuously drives differentiation in community composition throughout the monitoring period. During the heating stage, with an R-value of 0.609, the T400 samples were already clearly segregated from the other groups, suggesting that wood vinegar produced at 400 °C can establish a distinct community structure at an early stage. In the high-temperature stage, the group separation reached its maximum (R = 0.858), with T400 exhibiting the most distinct boundary from the other treatments and almost no overlap, indicating the strongest explanatory and discriminatory power at this stage. Subsequently, during the cooling and maturation stages (R = 0.501 and 0.384, respectively), increased overlap of sample points and reduced intergroup distances on the PCA plots were observed. This suggests that the differentiation effect of treatments on microbial communities gradually diminishes, with community structures converging toward a relatively stable state, likely reflecting microbial environmental adaptation. Over time, through metabolic adjustment and ecological niche reconstruction, microbial communities adapt to the environment, resulting in attenuated differences and stabilization, which underscores their intrinsic self-regulatory capacity (Zhong et al., 2020). Nevertheless, T400 and other wood vinegar treatments remained distinguishable from the control, demonstrating that wood vinegar exerts a sustained influence on microbial community structures within the compost matrix.

**FIGURE 9:**
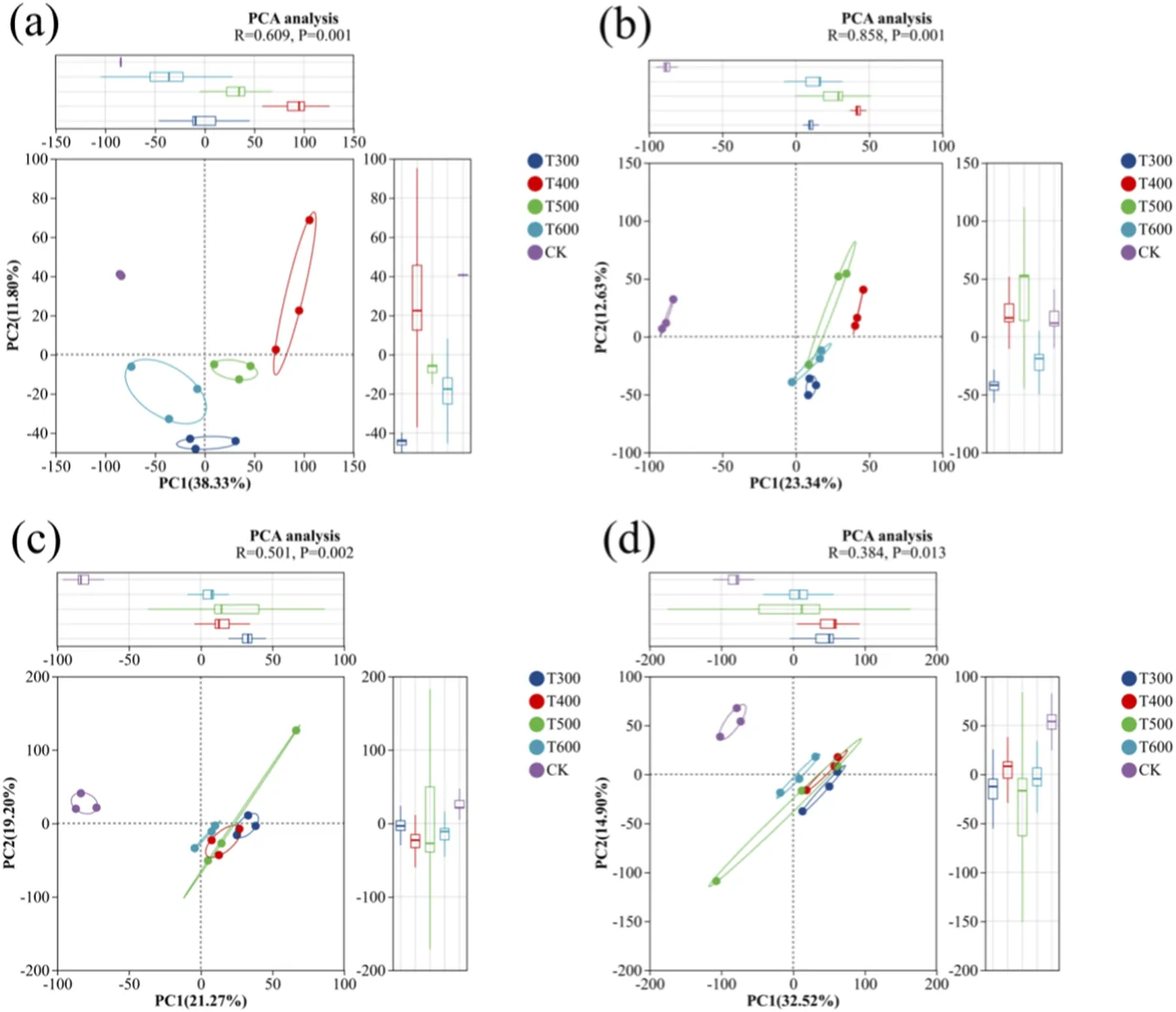
Principal component analysis (PCA) of microbial β-diversity during composting under different treatments (CK, T300, T400, T500, and T600). **(a)** Initial stage, **(b)** Thermophilic stage, **(c)** Cooling stage, and **(d)** Maturation stage. The percentage of variance explained by each principal component (PC1 and PC2) is indicated on the axes.

#### 3.5.2 Succession characteristics of bacterial and fungal communities

The addition of wood vinegar modulated both the composition and abundance of dominant bacterial and fungal taxa throughout the composting process (Figure 10).

**FIGURE 10:**
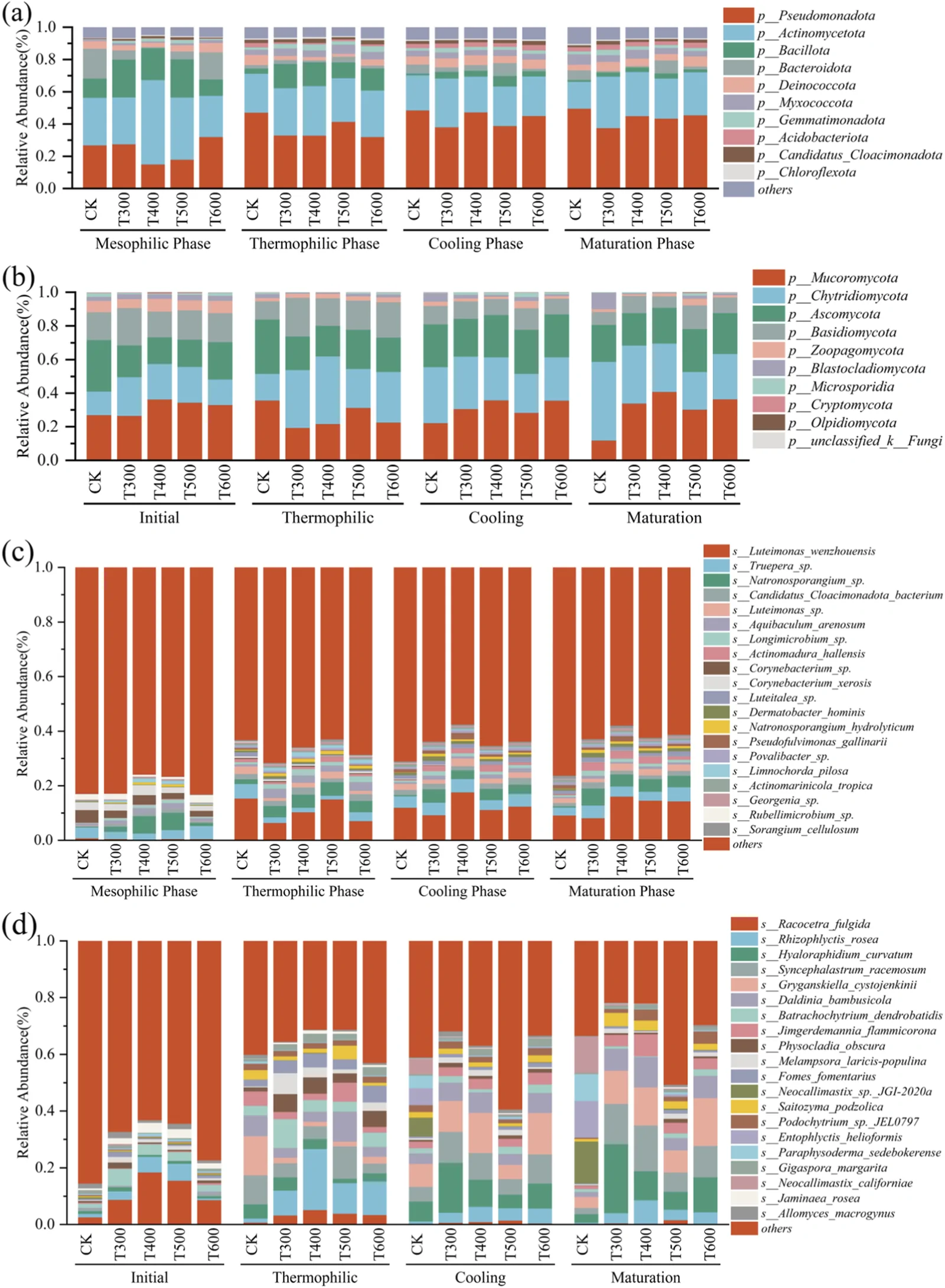
Microbial community composition during composting under different treatments (CK, T300, T400, T500, and T600). **(a)** Bacterial community at the phylum level (top 10 phyla). **(b)** Fungal community at the phylum level (top 10 phyla). **(c)** Bacterial community at the species level (top 20 species). **(d)** Fungal community at the species level (top 20 species). The relative abundance is expressed as a percentage for each stage: Initial, thermophilic, cooling, and maturation.

Bacterial communities: During the heating stage, bacterial communities at the phylum level were primarily composed of Pseudomonadota, Actinomycetota, and Bacillota (Figure 10a). In the T400 treatment, Actinomycetota dominated, representing 52.36% of the community. This phylum contributes to rapid degradation of readily available organic matter, releasing heat and initiating partial lignocellulose decomposition, thereby accelerating the compost temperature rise and establishing a critical foundation for a successful transition to the thermophilic maturation stage (Wang et al., 2016). Species-level analysis revealed that the dominant bacteria at this stage primarily included *Luteimonas wenzhouensis, Luteimonas sp., Longimicrobium sp., Truepera sp.*, and *Natronosporangium sp.* Among these, *Luteimonas wenzhouensis* and *Luteimonas sp*. exhibited higher relative abundance under T400 treatment. This indicates that during the high-temperature phase under T400 treatment, the dominant bacterial community became more concentrated, which may be related to the relatively stable microbial community during the high-temperature stage of the compost pile under this treatment.

During the composting high-temperature stage, the relative abundance of Bacillota in CK, T300, T400, T500, and T600 was 3.2%, 15.02%, 14.81%, 9.81%, and 13.73%, respectively. Wood vinegar selectively enriched thermotolerant microbial taxa with strong degradation capacity. This phylum is mainly composed of thermophilic spore-forming *Bacillus*, which can efficiently degrade proteins, starch, and hemicellulose, thereby sustaining high composting temperature and promoting humification (Chen et al., 2023; Finore et al., 2023). Species-level analysis revealed that the dominant bacteria at this stage primarily included *L. wenzhouensis*, *Luteimonas sp.*, *Longimicrobium sp.*, *Truepera sp.*, and *Natronosporangium sp.* Among these, *Luteimonas wenzhouensis* and L*uteimonas sp.* exhibited higher relative abundance under T400 treatment. This indicates that during the high-temperature phase under T400 treatment, the dominant bacterial community became more concentrated, which may be related to the relatively stable microbial community during the high-temperature stage of the compost pile under this treatment. In the cooling and maturation stages, Pseudomonadota and Actinomycetota remained the dominant bacterial phyla, jointly driving late-stage organic matter stabilization and humification.

Fungal communities: Across all treatments, the dominant fungal phyla were Mucoromycota, Chytridiomycota, Ascomycota, and Basidiomycota. During the heating stage, Mucoromycota accounted for 36.30% of the T400 community, surpassing CK (26.93%), T300 (26.49%), T500 (34.39%), and T600 (32.98%). The enhanced abundance is attributable to the strong degradative capacity of Mucoromycota toward starch, simple sugars, and hemicellulose, thereby promoting rapid transition into the high-temperature stage (Li et al., 2020). The species-level results further demonstrated that *Racocetra fulgida* exhibited significantly higher relative abundance in the T400 treatment during the warming period compared to other treatments, while *Jaminaea rosea* and *Paraphysoderma sedebokerense* also showed elevated abundance, indicating faster establishment of early dominant fungi under the T400 treatment. Combined with the phylum-level results, this phenomenon may be attributed to the enhanced response of fungal communities to easily degradable organic components during the warming phase under the T400 treatment.

Following the high-temperature stage, wood vinegar addition facilitated the enrichment of Chytridiomycota in all experimental treatments, enhancing the decomposition of lignin and complex cellulose. This supports continuous organic substrate transformation while stabilizing microbial communities during the thermophilic stage (Hanrahan-Tan et al., 2023).

#### 3.5.3 KEGG level 3 functional pathway enrichment analysis

KEGG level-3 pathway enrichment analysis revealed pronounced differences in metabolic functional potential metabolic functions across the treatments (Figure 11). Across the entire composting process, treatments exhibiting stronger humification effects were typically accompanied by the coordinated upregulation of multiple key metabolic pathways, indicating greater microbial potential for substrate transport, carbon and nitrogen metabolism and organic acid transformation. Carbon metabolism and glyoxylate and dicarboxylate metabolism were significantly enriched in the CK versus T300 (*), CK versus T400, and CK versus T600 comparisons, indicating that the addition of an appropriate dose of wood vinegar can confer microbes higher potential to sustain central carbon metabolism and degrade refractory substrates (Yang et al., 2023). Compared with other treatments, CK versus T400 also exhibited significant enrichment in valine, leucine, and isoleucine degradation pathways, suggesting that this treatment effectively has stronger genetic potential to decompose protein organic matter and supply metabolic carbon and energy skeletons (Yao et al., 2024).

**FIGURE 11:**
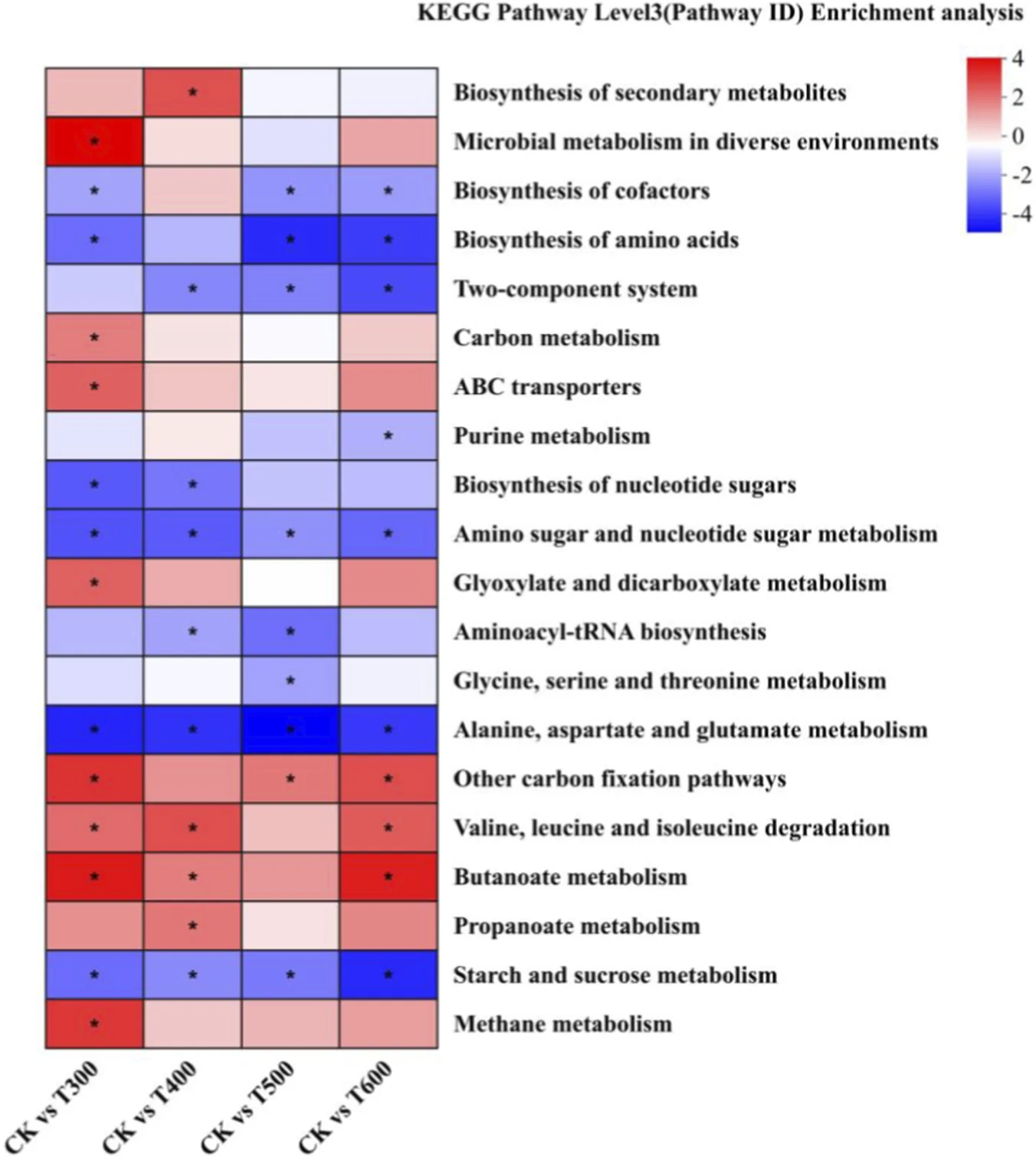
KEGG Pathway Level 3 (Pathway ID) enrichment analysis of the top 20 functional categories based on differential abundance. The x-axis shows the treatment comparisons (CK vs T300, CK vs T400, CK vs T500, and CK vs T600), and the y-axis lists KEGG Level 3 pathways, and the color scale indicates the enrichment of each pathway (red indicates enrichment in the treatment group, blue indicates enrichment in the control group). Stars indicate significantly enriched pathways, with an absolute pathway reporter score ≥ 1.65.

Additionally, butanoate and propanoate metabolism were significantly enriched in the T400 group, highlighting an enhanced microbial potential for the transformation and reutilization of organic acid intermediates. This likely represents a potential pathway to reduce organic acid accumulation and stabilize compost systems (Zhang et al., 2024).

By contrast, although T500 and T300 treatments exhibited some enrichment in specific degradation-related pathways, their metabolic potential advantages were less comprehensive than those observed in T400. T500 showed modest enhancement in branched-chain amino acid degradation and butanoate metabolism but comparatively weaker enrichment in core carbon metabolism and propanoate metabolism. T300 demonstrated stronger enrichment in carbon metabolism, ABC transporters, and diverse microbial metabolism pathways during the initial substrate utilization stage, yet displayed limited enhancement in pathways associated with organic acid turnover and late-stage stabilization, thereby constraining overall potential humification efficiency. In the T600 treatment, pathway enrichment was more dispersed, and synergistic effects were weak, while the control primarily enriched pathways involved in starch, sucrose, and amino sugar metabolism, reflecting utilization of easily degradable substrates without carrying the genetic potential to build high-efficiency decomposition metabolic networks. Collectively, coordinated activation of multifunctional pathways endows T400 with the greatest genetic potential for superior humification. It is important to emphasise that the contributions of functional pathways, as inferred from enrichment results, should not be equated with their actual activity or effectiveness in the processes of organic substrate degradation, intermediate metabolite transformation, and humus formation. Future research should aim to integrate metatranscriptomics, metabolomics, and key extracellular enzyme activities to dynamically analyse the expression and activation status of functional genes and metabolic pathways at various stages of composting. This approach will facilitate the identification of key functional microorganisms that drive organic carbon degradation and humus formation, thereby establishing a direct link between microbial genetic potential, functional expression, and the processes of carbon conversion and humification in composting.

### 3.6 Structural equation analysis

From a general statistical perspective, temperature shows a positive correlation with humic acid (HA) accumulation, suggesting that increased temperature tends to facilitate HA generation (Figure 12). Higher temperatures can enhance the metabolic activity of functional microorganisms, accelerate the degradation and humification of organic matter, and thereby contribute to the formation of humic substances (Gao et al., 2024). In contrast, microbial abundance (calculated as the cumulative abundance of microbial taxa excluding the top ten most abundant species) presents a negative statistical correlation with HA content. This negative correlation may indirectly reflect the relative enrichment of dominant functional microbial communities and the reduction of non-dominant microbial populations, which is likely a key community structural characteristic driving efficient HA synthesis. Combined with the microbial succession results in Section 3.5.2, this negative relationship does not indicate that overall microbial activity inhibits HA formation. Instead, the screening effect of wood vinegar increases the proportion of key functional dominant phyla including Actinobacteriota, Firmicutes, Mucoromycota, and Chytridiomycota, forming a concentrated and stable microbial community structure. The competitive suppression of non-dominant microbial taxa by dominant functional microbes further enables the directional decomposition of lignocellulose and the metabolism of humification precursors, thereby continuously promoting the aromatization, polymerization and accumulation of HA.

**FIGURE 12:**
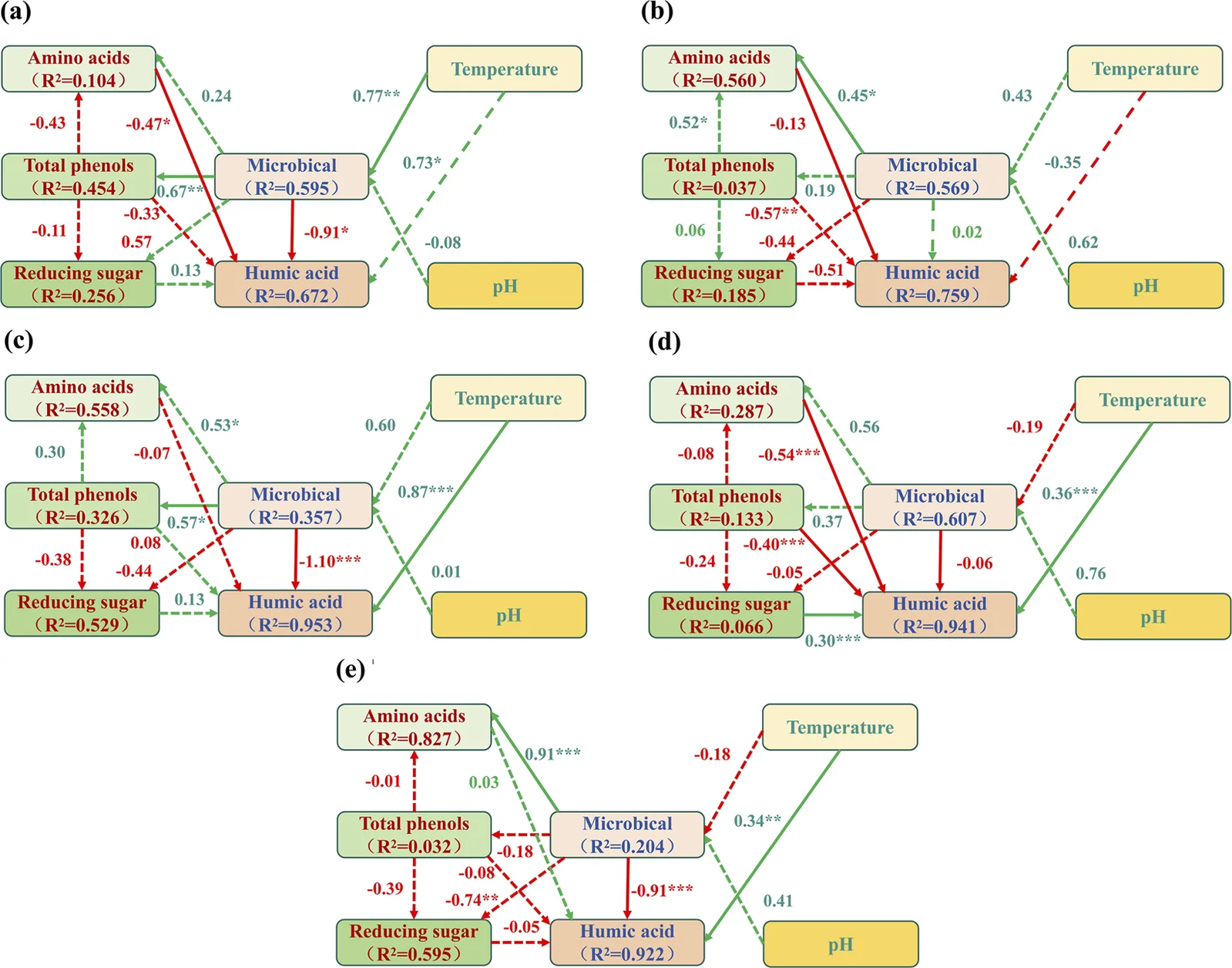
Structural equation models (SEMs) depicting relationships among amino acids, total phenols, reducing sugars, microbial factors, temperature, pH, and humic acid (HA) under **(a)** CK, **(b)** T300, **(c)** T400, **(d)** T500, and **(e)** T600 treatments. Green and red arrows indicate positive and negative standardized path coefficients, respectively. Solid and dashed arrows indicate significant and non-significant paths. Numbers adjacent to arrows are standardized path coefficients; values in boxes are R^2^. *P < 0.05, **P < 0.01, ***P < 0.001.

In terms of group-specific structural equation model results, the T400 group exhibited a significant positive path effect of temperature on HA (0.87***) and a significant negative path effect of microbial factors on HA (−1.10***). The combination of the two factors formed the most prominent humification-promoting correlation path at the statistical level, showing the optimal humification promotion effect among all groups. For the T500 group, HA accumulation was positively regulated by temperature (0.36***) and reducing sugars (0.30***), and negatively correlated with amino acids (−0.54***) and total phenols (−0.40***). Statistically, these results indicate that the humification process of the T500 group was mainly supported by substrate consumption and transformation, as well as the synergistic effect of temperature and carbon sources. Although the overall humification effect was significant, the degree of microbial community dominance was lower than that in the T400 group. In the T300 group, HA variationwas primarily regulated by a single factor, total phenols (−0.57**), with only a weak correlation observed between microbial characteristics and HA. The low enrichment level of dominant microbial communities in this group led to a weaker humification promotion effect compared with the T400 and T500 groups. Similar to the T400 group, both the T600 and CK groups showed significant positive temperature correlation and significant negative microbial correlation with HA generation, yet the strength of these path correlations was inferior to that of the T400 group. Overall, structural equation model analysis suggests that the superior HA accumulation performance of the T400 group can be attributed to the dual correlational pathways of enhanced temperature effect and optimized microbial community structure. Temperature and microbial community structure differences are the key potential dominant factors regulating HA accumulation during composting, enabling the T400 group to present a significantly better humification promotion potential than other treatment groups.

## 4 Conclusion

This study compared the effects of cotton-stalk-derived wood vinegar produced at different pyrolysis temperatures on composting thermal dynamics, humification, precursor transformation, and microbial community succession. The results indicated that the T400 treatment exhibited the optimal performance in terms of heating rate, duration of the high-temperature stage, and peak temperature (60–65 °C), providing a stable and efficient thermal environment for the humification reaction. Regarding humification effects, the T400 group demonstrated the most significant accumulation of HA, with increases of 14% and 28% over the CK group on the 10th and 20th days, respectively. The conversion process from FA to HA was the smoothest, and the overall humification-promoting effect was ranked as T400 ≈ T500 > T300 > T600 > CK. Precursor substance utilization analysis further revealed that in the T400 group, the correlation network between polyphenols, amino acids, reducing sugars, and HA was the most coordinated, reflecting an efficient synergistic transformation mechanism. Spectroscopic evidence (FTIR and 3D-EEM) consistently demonstrated that the T400 group exhibited the highest degree of aromatization, a leading humification index (HFRI), and a more stable humus structure.

At the same time, microbial diversity analysis indicated that the T400 treatment caused a slight decline in bacterial and fungal alpha-diversity during the high-temperature stage. Nevertheless, this treatment selectively enriched core humification-related functional microorganisms, resulting in a concentrated microbial community structure with distinct functional differentiation, which provided solid biological support for the humification process.

In conclusion, T400 emerged as the optimal experimental group, balancing the maintenance of microbial diversity with the enhancement of humification efficiency. Moderate high temperatures significantly promoted the transformation of organic precursors.

## Data Availability

The original contributions presented in the study are publicly available. This data can be found here: NCBI BioProject, accession number PRJNA1540857.
