# Supplementary material for: Precursor transformation and microbial responses during compost humification regulated by wood vinegar produced at different pyrolysis temperatures
Source: Front Chem. 2026 Oct 21;14:1912238. doi: 10.3389/fchem.2026.1912238 (PMC13731379; doi:10.3389/fchem.2026.1912238)
Supplement: Supplementary file 1 [file DataSheet1.docx]

**Supplementary Materials**

In this experiment, swine manure was collected from a farm near the 12th Regiment, air-dried, and crushed into uniform small pieces (diameter < 0.5 cm). Wheat straw was collected from the Animal Science Experimental Base of Tarim University. Cotton stalks were obtained from the 12th Regiment and processed using a pyrolysis device at 300, 400, 500, and 600 °C to obtain cotton-stalk pyroligneous liquor at the corresponding temperatures. All raw materials were thoroughly mixed. The basic physicochemical properties of the initial materials are presented in Table 1.

*Table 1. Basic physicochemical properties of the raw materials*

| Material | pH | Moisture content (%) | TN (g/kg) | C/N | TC (g/kg) |
| --- | --- | --- | --- | --- | --- |
| Swine manure | 6.10 ± 0.13 | 45.48 ± 0.25 | 16.50 ± 0.31 | 12.69 | 209.31 ± 3.08 |
| Wheat straw | 6.82 ± 0.10 | 2.92 ± 0.20 | 6.80 ± 0.31 | 46.78 | 318.11 ± 1.78 |
| Pyroligneous liquor (300 °C) | 4.21 ± 0.05 | / | 8.95 ± 0.04 | 51.81 | 463.73 ± 3.08 |
| Pyroligneous liquor (400 °C) | 4.32 ± 0.04 | / | 8.41 ± 0.03 | 57.77 | 485.85 ± 5.18 |
| Pyroligneous liquor (500 °C) | 4.58 ± 0.03 | / | 8.15 ± 0.06 | 62.59 | 510.17 ± 6.38 |
| Pyroligneous liquor (600 °C) | 4.66 ± 0.06 | / | 8.55 ± 0.05 | 63.19 | 540.29 ± 3.58 |

Specifically, the C/N ratio of each raw material was first determined or obtained from preliminary characterization data. The required mixing proportion was then calculated according to the mass balance of total organic carbon and total nitrogen, using the following equation:

$$C/N=\frac{\sum(M_{i}\times C_{i})}{\sum{(M}_{i}\times N_{i})}$$

Where M_i_ , C_i_ , N_i_ represent mass, its carbon and nitrogen contents, respectively. Based on this calculation, carbon-rich materials were added to increase the C/N ratio when the nitrogen content was relatively high, whereas nitrogen-rich materials were supplemented when the C/N ratio was above the target range. The final initial C/N ratio of the composting mixture was adjusted to approximately 25–30:1 before composting, which is generally considered suitable for microbial activity and efficient organic matter degradation.

Supplementary GC–MS results for the pyroligneous liquor are presented below. According to the characterization results obtained in this study (Table 2), when the pyrolysis temperature increased from T300 to T600, the composition of the pyroligneous liquor exhibited a pronounced redistribution pattern. Phenolic compounds peaked within the intermediate-temperature range, increasing from 29.09% at T300 to 34.64% at T400, and then declining to 27.16% and 22.74% at T500 and T600, respectively. This trend is consistent with the current understanding that lignin decomposition in the intermediate-temperature range generates phenolic products, whereas at higher temperatures these compounds may undergo further secondary cracking, deoxygenation, or condensation/polymerization, thereby reducing their relative abundance in the liquid phase. Comparative GC–MS studies across multiple temperature ranges have likewise shown that the composition of aromatic oxygenated compounds such as phenolics in pyroligneous liquor changes substantially depending on the temperature window.

Carbonyl compounds and esters were markedly enriched at intermediate-to-high temperatures. Table 2 shows that ketones increased from 11.49% at T400 to 20.75% at T500 and then declined to 14.07% at T600; similarly, esters reached their highest proportion at T500 (19.33%) and remained relatively high at T600 (16.03%). These results suggest that, with increasing temperature, polysaccharide decomposition and rearrangement more readily generate carbonyl compounds, while esterification and other secondary reactions among acidic and alcoholic components are enhanced, thereby increasing the proportions of ketones and esters in the intermediate- and high-temperature samples. These observations are in agreement with the general conclusion from the literature that the relative abundance of ketones increases above 350 °C.

Aldehydes and N-containing heterocyclic compounds generally showed a decreasing trend or a decrease followed by stabilization. Aldehydes were relatively abundant at T400 (8.97%) but decreased to 4.05% and 4.68% at T500 and T600, respectively. N-containing heterocyclic compounds declined from 7.33% at T300 to 3.09% at T500, followed by a slight increase to 3.21% at T600. This phenomenon can generally be interpreted in light of the greater susceptibility of relatively unstable small molecules to further transformation or cracking under higher-temperature conditions, which is also consistent with published statistical results showing a relative decrease in acids and aldehydes at elevated temperatures. It is noteworthy that the acid fraction in Table 2 did not change monotonically: it decreased from 3.86% at T300 and 4.14% at T400 to 2.67% at T500, but then increased to 8.01% at T600. This intermediate-temperature decrease followed by a high-temperature increase indicates that acidic components are jointly controlled by multiple factors. On the one hand, acids may decrease at intermediate-to-high temperatures owing to decarboxylation, cracking, and related reactions. On the other hand, at still higher temperatures, certain secondary cracking pathways may increase the formation of specific organic acids or facilitate their transfer into the condensable liquid phase, leading to a rebound in total relative acid content. Studies of pyroligneous liquor derived from other feedstocks (e.g., walnut shells) have similarly shown that temperature can markedly alter the proportions of acids, phenols, ketones, and other components, resulting in a complex relationship between acidity and acid content.

*Table 2. Relative proportions of chemical classes in cotton-stalk pyroligneous liquor determined by GC–MS (%)*

| Class | T300 | T400 | T500 | T600 |
| --- | --- | --- | --- | --- |
| Amines | 2.71 | 1.48 | 0.35 | 2.36 |
| Alcohols | 9.99 | 9.07 | 12.95 | 12.08 |
| Phenols | 29.09 | 34.64 | 27.16 | 22.74 |
| N-containing heterocycles | 7.33 | 6.21 | 3.09 | 3.21 |
| Aldehydes | 5.71 | 8.97 | 4.05 | 4.68 |
| Acids | 3.86 | 4.14 | 2.67 | 8.01 |
| Ketones | 10.88 | 12.96 | 19.33 | 16.03 |
| Amides | 4.73 | 6.01 | 6.16 | 6.39 |
| Esters | 12.54 | 11.49 | 20.75 | 14.07 |
| Not belonging to the above nine classes | 13.16 | 5.00 | 3.48 | 10.42 |

Furthermore, the similarity index (SI) distribution obtained from GC–MS library retrieval provided indirect evidence for increased compositional complexity in the high-temperature samples (Table 3). As the temperature increased from T300 to T600, the proportion of compounds with SI ≥ 800 decreased from 24.80% to 13.53%, whereas the proportion with SI < 700 increased from 36.33% to 56.42% and remained high at T500–T600 (54.59%–56.42%). The SI, or match factor, is an indicator of identification confidence based on spectral library matching; lower values generally suggest insufficient library coverage, co-elution interference, or more complex target structures with weaker similarity to the reference spectra. This result may be attributed to the greater prominence of secondary cracking, deoxygenation, and condensation/polymerization reactions at higher pyrolysis temperatures, which generate more complex components (or newly formed compounds) in the pyroligneous liquor that cannot be assigned with high confidence using conventional spectral libraries. This interpretation is consistent with recent studies indicating that the composition of pyroligneous liquor is highly dependent on pyrolysis conditions and that higher-temperature ranges may increase compositional complexity and the difficulty of standardization.

*Table 3. Distribution of similarity index (SI) values from GC–MS library retrieval*

| Group | T300 | T400 | T500 | T600 |
| --- | --- | --- | --- | --- |
| SI ≥ 800 | 24.80 | 19.76 | 14.38 | 13.53 |
| 700 ≤ SI < 800 | 38.87 | 51.42 | 31.03 | 30.04 |
| SI < 700 | 36.33 | 28.82 | 54.59 | 56.42 |

*Table 4. Response order of functional groups for CK and each treatment group (wavenumber unit: cm^-1^)*

| group | 1 | 2 | 3 | 4 | 5 | 6 | 7 | 8 | 9 | 10 | 11 | 12 |
| --- | --- | --- | --- | --- | --- | --- | --- | --- | --- | --- | --- | --- |
| CK | 1540 | 1720 | 1630 | 1356 | 2829 | 775 | 2717 | 2920 | 3404 | 1420 | 1270 | 1064 |
| T300 | 1064 | 1630 | 1356 | 1540 | 775 | 2829 | 2920 | 1420 | 1270 | 1720 | 3404 | 2717 |
| T400 | 1064 | 1270 | 1420 | 1540 | 1720 | 2920 | 3404 | 1630 | 2717 | 1356 | 2829 | 775 |
| T500 | 1356 | 775 | 1540 | 1630 | 2829 | 1720 | 2717 | 1420 | 2920 | 1270 | 3404 | 1064 |
| T600 | 1064 | 3404 | 1270 | 1420 | 2920 | 1630 | 1540 | 1720 | 1356 | 2829 | 2717 | 775 |
